# Enhanced dissolution of arsenic in anaerobic soils upon organic amendment application: acid detergent-soluble organic matter as a potential indicator

**DOI:** 10.1038/s41598-022-27325-1

**Published:** 2023-01-05

**Authors:** Aomi Suda, Koji Baba, Gen Sakurai, Manami Furuya, Noriko Yamaguchi

**Affiliations:** 1 Institute for Agro-Environmental Sciences, NARO, Kannondai 3-1-3, Tsukuba, Ibaraki 305-8604 Japan; 2 Research Center for Advanced Analysis, NARO, Kannondai 3-1-3, Tsukuba, Ibaraki 305-8604 Japan

**Keywords:** Geochemistry, Element cycles

## Abstract

Application of organic amendments (OAMs) often enhances arsenic (As) dissolution in paddy soils. Therefore, understanding the properties of OAMs that determine the extent of As dissolution is essential for appropriate soil management. Since As dissolution increases with decrease in soil redox potential caused by microbial respiration, the decomposability of OAMs might be a critical factor controlling As dissolution in amended soils. We hypothesized that contents of acid detergent-soluble organic matter (ADSOM, mainly composed of non-fiber organic matter and hemicellulose) in OAMs can help estimate the potential of OAMs in accelerating As dissolution in soils with added OAMs. Therefore, two contrasting soil types, Andosol and Fluvisol, were mixed with 24 different OAMs and subjected to anaerobic incubation for 14 weeks. Changes in soil Eh and dissolved As contents were monitored throughout the incubation period, and As species in solid phases and ferrous iron (Fe(II)) contents in soils were measured after 2 and 6 weeks of incubation. The higher the ADSOM content in soils with OAMs, the higher the dissolved As contents in soils and the lower the Eh values. Dissolved As also positively correlated with the proportion of As(III) in solid phases and Fe(II) content after 2 and 6 weeks of incubation, indicating that decomposition of ADSOM led to reducing soil conditions, thereby promoting the reduction of As(V) and As-bearing Fe oxides and subsequent As dissolution. The results were consistent between the two types of soils, despite dissolved As content in the Andosol being two orders lower than that in Fluvisol. This is the first study to demonstrate that ADSOM can be a prominent indicator of the potential of OAMs, for promoting As dissolution, when applied to paddy soils.

## Introduction

Arsenic (As) ubiquitously exists in soils as inorganic As and organic species. Soils in rice paddies undergo flooding during part of the cultivation period, which creates conducive conditions for rice plants to absorb As from the soils^1^. Prolonged soil flooding causes an increase in dissolved As concentrations in paddy soils. Respiration of soil microorganisms decreases soil redox potential, resulting in As(V) reduction to As(III) in soils. Both As(V) and As(III) are sorbed on a wide range of soil minerals, such as Fe oxides, aluminum (Al) minerals, and clay minerals^2–4^. However, As(III) sorption on these minerals is much lower than that of As(V)^2,5,6^, although comparable or greater amounts of As(III) than As(V) are sorbed on Fe oxides at near neutral pH^7,8^. In addition, co-existing dissolved organic matter (DOM) and dissolved anions increase As dissolution from soils and related materials by competing for sorption sites with As^9–11^, and this more significantly occurs for As(III) than As(V) in weakly acidic to neutral pHs^8,10^. Thus As(V) reduction to As(III) results in the dissolution of As from solid phases in soil. The release of As from anaerobic soils is also considered to arise from the reductive dissolution of As-bearing Fe oxides, as As and Fe are closely and positively related in soil solutions^12,13^. However, recent studies have demonstrated that reducing As-bearing Fe oxides does not always cause an increase in dissolved As content because released As is incorporated into newly formed secondary Fe phases^14,15^; nevertheless, prolonged reduction and dissolution of Fe oxides eventually cause release of As into the solution phase in soil^16^.

The supplemental use of organic amendments (OAMs) in paddy soils have been recently re-evaluated to improve their chemical properties and nutrient status for rice cultivation^17,18^. The use of OAMs in paddy fields is beneficial as they help either reduce the need for or replace chemical fertilizers; however, their ability to solubilize As is a matter of concern. Although OAMs can accelerate reduction processes and increase DOM content in soils, the application of OAMs decreases available As content, thereby lowering As concentrations in rice plants^19,20^. This may occur because As is fixed with humic substances or complexed with DOM^21,22^, whereas —and this probably happens more frequently— OAM application dissolves more As into the solution phase of anaerobic soils and enhances As uptake by rice plants^23,24^. Suda and Makino^25^ demonstrated that the increase in As dissolution in anaerobic soils with the application of OAMs could be explained by the acceleration of soil reduction closely associated with bio-decomposability of the applied OAMs (i.e., As detachment induced by the reduction of As(V) and As-bearing Fe oxides), rather than by the increase in competing substance contents for sorption sites.

Thus, the elucidating the bio-decomposability of each OAM is crucial for determining their relative As dissolution potential in anaerobic soils with OAMs, although the actual amount or rate of As dissolution is dependent on the soil properties and total As contents. Nonetheless, most studies on As dissolution in soils with OAMs have not presented detailed OAM properties, and only few studies have focused on their bio-decomposability and As dissolution in applied soils^25,26^. The carbon to nitrogen ratio (C/N) has been frequently considered a factor affecting the bio-decomposability of OAMs^27–29^: for instance, Suda and Makino^25^ demonstrated that the application of OAMs with low C/N led to a rapid decrease in soil redox potential (i.e., rapid decomposition, and thereby accelerated As dissolution from anaerobic soils). However, they used 15 well-composted and well-fermented OAMs, and excluded immature (i.e., not composted or fermented well) OAMs. Traditionally, many kinds of the latter have been applied to paddy soils, and some of them have had both large C/N and high bio-decomposability. Thus, C/N seems to be less of a comprehensive indicator of bio-decomposability and enhancement potential for As dissolution, and an alternative indicator applicable for the wide range of OAMs is required. Recently, acid detergent analysis has been used to predict the decomposition characteristics of manure and compost in applied soils^30,31^. Acid detergent soluble organic matter (ADSOM) is mainly composed of non-fiber organic substances and hemicellulose, closely agrees with the organic matter decomposable within 3 months^30,31^. Since ADSOM in OAMs is subject to microbial decomposition, increasing amounts of ADSOM would accelerate reducing conditions in flooded soils, thereby enhancing As dissolution. However, the relationship between ADSOM in OAMs and As dissolution from anaerobic soils with OAMs are yet to be established.

The objectives of the present study were to reveal: (1) how different levels of ADSOM in OAMs influence solid-state reduction of As(V) to As(III) and dissolution of As from soils during the development of reductive conditions, (2) the relation between ADSOM and As dissolution in two contrasting soils, Fluvisols and Andosols, and (3) the expediency of ADSOM to indicate if OAM application causes increased dissolution of As from the soil under flooded conditions, based on comparisons of 24 different OAMs. Through these objectives, our study hopes to facilitate selection of appropriate OAMs to be applied to paddy fields to help minimize As exposure to rice plants.

## Materials and methods

### Preparation and analysis of soil samples

We collected two types of soils from plow layers in unpolluted paddy fields at the Institute for Agro-Environmental Sciences, National Agriculture and Food Research Organization (Kanto region, Japan). One of the soil samples was collected from the plow layer of a paddy field (36° 1′ 27.696″N, 140° 6′ 28.836″E) that had been transported from a different field categorized as Fluvisol (hereafter referred to as “F-soil”). Fluvisols represent young soils in fluvial deposits. The other, Andosol (referred as “A-soil”) (36° 1′ 27.6594″N, 140° 6′ 23.004″E), is one of the volcanic ash soils and is widely distributed throughout Japan. The collected moist soils were passed through 2-mm mesh sieves and stored at 5 °C until use. Their aliquots were air-dried at room temperature and used for further analyses of several soil properties by employing appropriate methods (see Supplementary Files).

### Preparation and analysis of organic amendments

General information on the examined OAMs is shown in Table 1. The OAMs were obtained commercially or made in or collected from the fields in the research institute. The OAMs were then pulverized using a Wiley mill after being air-dried at room temperature, and finely ground with zirconia balls using a Shake Master Neo (Bio-Medical Science, Tokyo, Japan). Ground samples were stored at 5 °C until their use for chemical analyses and incubation experiments.

**Table 1:** Information of examined organic amendments (OAMs) including acid detergent soluble organic matter (ADSOM) and OAM carbon to nitrogen ratio (C/N).

ID	Name	Raw material	ADSOM (g kg^−1^)	C/N (g g^−1^)
**Plant-based OAM**				
No. 1	Bark compost	Bark	− 44.9	50.5
No. 2	Leaf compost	Leaf, branch	22.2	22.5
No. 3	Rice straw compost	Rice straw, rice husk	79.9	12.1
No. 4	Rice husk compost	Rice husk, rice straw, sawdust	126	16.9
No. 5	Sawdust compost	Sawdust, cow dung, bark	155	16.4
No. 6	Branch compost	Branch, cow dung	95.2	22.0
No. 7	Fermented rapeseed waste	Rapeseed-oil waste, char	318	8.80
No. 8	Fermented rice bran	Rice bran, tofu waste, rapeseed-oil waste	613	9.83
No. 9	Rice straw	Rice straw	454	90.9
No. 10	Rice husk	Rice husk	239	101
No. 11	Wheat straw	Wheat straw	352	47.5
No. 12	Hairy vetch	Hairy vetch	463	14.8
No. 13	Clover	Clover	591	13.9
No. 14	Rapeseed waste	Rapeseed-oil waste	706	6.70
**Animal-based OAM**				
No. 15	Cow manure-1	Cow dung, woody waste, straw	162	11.1
No. 16	Cow manure-2	Cow dung, sawdust	162	15.4
No. 17	Horse manure	Horse dung, straw	96.0	18.4
No. 18	Mixed manure	Livestock and plant processing waste, poultry dung	430	8.82
No. 19	Fermented swine dung-1	Pig dung	393	9.11
No. 20	Fermented swine dung-2	Pig dung	418	9.13
No. 21	Fermented chicken dung-1	Chicken dung	489	7.50
No. 22	Fermented chicken dung-2	Chicken dung	344	9.76
No. 23	Fermented chicken dung-3	Chicken dung	334	10.1
No. 24	Fermented chick dung	Chick dung	527	10.2

The extraction of pseudo-total As in each OAM was performed using acid digestion with a mixture of HNO_3_ and HClO_4_ on a hot plate, and it was followed by quantification using inductively coupled plasma mass spectrometry (ICP-MS; NexION300X, PerkinElmer, Waltham, MA, USA). C and N contents in each OAM were determined using an NC analyzer (Sumigraph NC-900; Shimadzu), and ADSOM in OAM was determined using the following procedure^32^: 1 g of OAM was mixed with 100 mL of acid detergent solution which is a mixed solution of 20 g of cetyltrimethylammonium bromide and 1 L of 0.5 mol L^−1^ sulfuric acid. The mixture was heated in boiling water for 1 h and filtered using filter paper in a Buchner funnel, and the residue was washed thoroughly with hot water and acetone under reduced pressure. The washed residue on the filter was oven-dried and weighed (A: acid-detergent insoluble organic matter and silica), and then transferred into a ceramic crucible for incineration at 600 °C for 2 h after preliminary incineration on a hot plate. Then, the weight of the resulting white–gray powder on the crucible was weighed (B: silica). Each OAM was incinerated separately, and then this residue (C: crude ash, including silica), which possibly contained a small amount of residual organic carbon, was weighed. The amount of ADSOM was calculated using the following equation:

1 $$\mathrm{ADSOM}=1000-\left(\mathrm{A}-\mathrm{B}\right)-\mathrm{C } \, (\mathrm{g }\,{\mathrm{kg}}^{-1})$$

### Anaerobic soil incubation

Long-term soil incubation under anaerobic conditions was performed as described in our previous study^25^, with slight modifications. Each finely ground OAM (0.05 g, oven-dried basis) was collected in a 50 mL glass vial. Ultra-pure water and moist soil (10 g, oven-dried basis) was added to the vial to bring the total water volume to 30 mL. The ratio of OAMs to soil is equivalent to 5 Mg ha^−1^, assuming a plow layer depth of 10 cm with a bulk density of 1 g cm^−3^. The application rate of OAMs was within the range of the field-application rate of OAMs^33^, and was selected to compare the effects of decomposability of different types of OAMs based on their weight. A similar vial without any OAM was prepared as a control. After 2 min of nitrogen gas (N_2_) purging, the vial was sealed with an inner butyl rubber cap and an outer aluminum cap. The sealed vial was manually shaken and put in an incubator at 30 °C for specific incubation times (10 min [as initial status], 1, 2, 4, 6, 9 and 14 weeks). The vial was subjected to manual shaking and its position in the incubator changed after every 2 or 3 incubation days. Two vials were prepared for each treatment.

After the incubation, the soil solution in the vial was sampled using an assembled unit composed of a syringe, a 0.2-μm pore filter (DG2M- 330, Spectrum Laboratories, Inc., Rancho Dominguez, CA, USA), and a needle (NN-2360C, Terumo Corporation, Tokyo, Japan). The unit was purged with N_2_ before use to avoid oxygen contamination in the vial and exposure to the sampled soil solution. The incubated vial was manually shaken and centrifuged at 608 x*g* (2000 rpm) for 5 min to settle the solid phases. The needle of sampling unit was then inserted into the vial through the butyl rubber cap to aspirate and filter the supernatant solution. Approximately 4.5 mL of filtrate was mixed immediately with 0.5 mL of 1.6 mol L^−1^ HNO_3_ to prevent the changing of As species and the precipitation of Fe hydroxides in the solution.

An aliquot of the filtrated soil solution was used immediately for pH measurement with a portable pH meter (LAQUA Twin pH, Horiba, Kyoto, Japan). Except for the initial soils (i.e., after 10-min incubation), the Eh of the residual suspension was measured with a combined electrode composed of platinum and silver–silver chloride electrode assembly (InLab Redox Micro, Mettler Toledo, Switzerland) under an N_2_ atmosphere.

### Analysis of dissolved As and Fe in soils

Speciation of As in the soil solution was determined using high-performance liquid chromatography (HPLC)-ICP-MS (HPLC: PerkinElmer Flexar HPLC System) within 48 h after soil solution sampling. The standard As species that were measured included arsenite [As(III)], arsenate [As(V)], monomethylarsonic acid (MMA), dimethylarsinic acid (DMA), and arsenobetaine (AsB). In a few cases, unidentified species in small amounts were detected in soil solutions and were quantified using the standard curve for As(V). The limit of detection (LOD) was 0.28 and 0.11 μg kg^−1^, and the limit of quantification (LOQ) was 0.94 and 0.37 μg kg^−1^ for F-soil and A-soil, respectively. The total amount of dissolved As in soil solutions was calculated by adding the values for all species, including unidentified ones, higher than the LOD. When none of the species exceeded the LOD, the total As in the soils was fixed at 0 μg kg^−1^ in the data analysis. The concentration of dissolved Fe was measured via inductively coupled plasma-optical emission spectrometry (Agilent 700 Series, Agilent Technologies, Santa Clara, CA, USA). LOD was 0.011 mg kg^−1^ and LOQ was 0.036 mg kg^−1^. Moreover, for each dissolved As species and dissolved Fe, data lower than the LOD were reported as 0 mg kg^−1^, and those above LOD but below LOQ were reported as the calculated values. Further details are provided in the Supplementary Files.

### Speciation of arsenic in soil solid phases

For As speciation in solid phases, control soils and OAMs (2, 5, 9, 10, 13, 14, 19 and 23) soils were collected after 2- and 6-week anaerobic incubations. Soils without any OAMs were also sampled after a 10-min incubation to determine initial As speciation. Duplicates of the incubated soils in vials were transferred into a 50 mL polyethylene centrifuge tube and then mixed well under an N_2_ atmosphere. The suspension was centrifuged at 1700 x*g* (3500 rpm) for 20 min to settle the solid particles. After removing the supernatant, an aliquot of wet soil paste was immediately packed into a polyethylene bag under an N_2_ atmosphere, and it was kept frozen at − 28 °C until analysis. The As K-edge (11,867 eV) X-ray absorption near-edge structure (XANES) spectra of the soil paste was obtained with BL5S1 at the Aichi Synchrotron Radiation Center. Disodium arsenite diluted by boron nitride and arsenate sorbed on ferrihydrite were used as standard materials for As(III) and As(V), respectively, and orpiment (As_2_S_3_) was used as the standard for As bound to sulfurs, such as As sulfides, and to the sulfhydryl group in organic matter. None or negligible amounts of organic As were detected in the soil solutions we collected (as described later). In addition, the preliminary measurement of concentrated HNO_3_-extracted solutions in selected soil pastes showed that organic As in solid phases was negligible (Supplementary Fig. S1). Therefore, spectra data on the standards for organic As species were not included in the fitting calculation below. The XANES spectra of all standards, except for As(V), were collected in transmission mode, and fluorescence detection mode was adopted to obtain XANES spectra for soil pastes and reference As(V) using a 7-element silicon drift detector. The proportion of As species in soil pastes was calculated with a linear combination fitting (LCF) ranging from 11,850–11,900 eV. The analysis of obtained XANES data was carried out using Athena in the Demeter software package (ver. 0.9.25).

### Analysis of ferrous iron in soils

To quantify Fe(II) in incubated soils, control and OAMs (2, 5, 9, 10, 13,14, 19 and 23) soils were collected after 2 and 6 weeks of incubation. Fe(II) was extracted and measured using the protocol proposed by Kumada and Asami^34^, with slight modifications. Soil Fe(II) was extracted using acidified sodium acetate and its content was measured using colorimetric methods (see Supplementary Files).

### Data analysis

A Spearman’s rank correlation analysis was conducted to test the statistical significance of nonlinear relationships among ratio of cumulative dissolved As in soils (see below), ADSOM content and OAM C/N, As(III) proportion in soil solid phases, and Fe(II), dissolved As and Fe contents, and soil Eh. Replication was averaged before rank-correlation analysis because Spearman’s rank correlation analysis assumes independency of each data point. R software (ver. 3.3.2)^35^ including EZR on R commander (ver. 1.32) was used to perform the data analysis.

## Results and discussion

### Properties of soils and organic amendments

The chemical properties of our soil samples are listed in Supplementary Table S1. A-soil had characteristic features of allophanic Andosols, which generally contain large amounts of SOM, oxalate-extractable Al (Al_o_), and oxalate-extractable silicon. Since A-soil had a relatively small amount of pyrophosphate-extractable Al, the primary Al-bearing substances extracted with oxalate were allophane-like minerals, not Al-humus complexes. The amounts of oxalate-extractable Fe (Fe_o_) and dithionite-citrate-extractable Fe (Fe_d_) in A-soil were approximately three times larger than those in F-soil, indicating that A-soil had notably more abundant amorphous and crystalline Fe oxides than F-soil. F-soil pH was weakly acidic, and that of A-soil was nearly neutral. The textures of F- and A-soil were light clay and heavy clay, respectively, and total As contents in both soils were close to the average As content in Japanese paddy soils (9 mg kg^−1^)^36^.

ADSOM and C/N ranges were − 44.9 to 706 g kg^−1^ and 6.70 to 101 g g^−1^, respectively. In this case, it is important to note that inorganic components can exist as oxides or salts, and these impurities in the ash fraction from the aforementioned C residue (see methods) may cause the overestimation of inorganic contents. Therefore, the actual ADSOM content could be slightly higher than estimated by the Eq. (1) used to calculate ADSOM levels. OAMs rich in ADSOM tended to have low C/N values and, conversely, OAMs poor in ADSOM tended to have high C/N values (see Table 1). However, three fresh (i.e., undecomposed) plant materials, namely No. 19 (rice straw), No. 20 (rice straw), and No. 21 (wheat straw), had abundant ADSOM and high C/N values. Lastly, As contents in OAMs ranged from 0.07 to 3.11 mg kg^−1^ (Supplementary Table S2)_._

### As dissolution in soils applied with organic amendments

**Figure 1:**
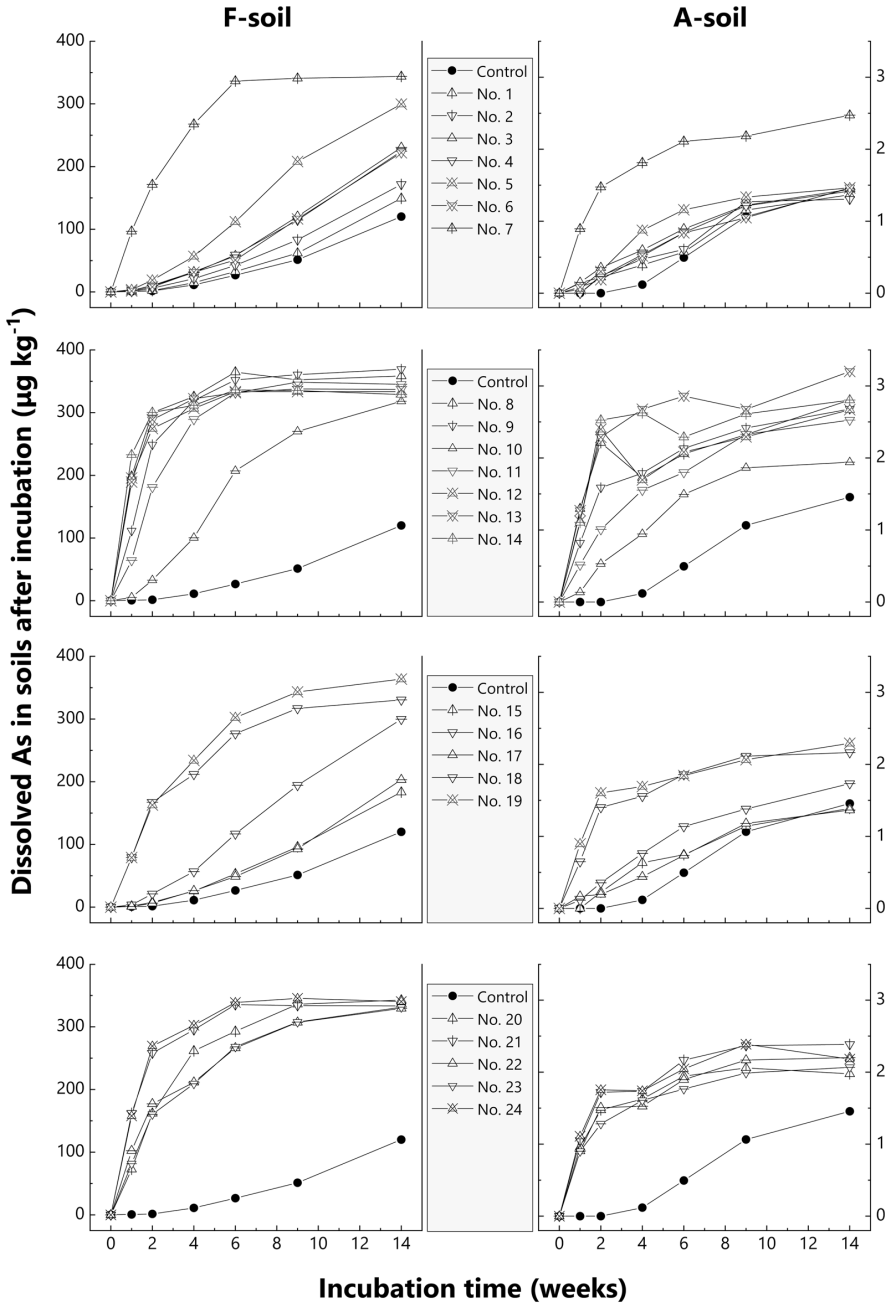
Time-course changes of dissolved As in F-soil and A-soil during anaerobic incubation. Panels on the left represent F-soils and those on the right, A-soils. Control is shown in all subfigures for comparison. Data are shown as the average of duplicates.

Throughout the anaerobic incubation period, soils mixed with OAMs more or less had higher dissolved As concentrations than control soils. The dissolution of As continued with time and the trend of additional As dissolution varied depending on the types of added OAMs (Fig. 1). For example, the addition of No. 1 (bark compost) and No. 15 (cow manure-1) slightly increased dissolved As, while No. 9 (rice straw) and No. 21 (fermented chicken dung-1) substantially increased the amount of dissolved As throughout the incubation period. Dissolution of As from soils mixed with some OAMs nearly reached a plateau at ~ 350 μg kg^−1^ for F-soil and ~ 2.5 μg kg^−1^ for A-soil before the end of incubation. Moreover, dissolved As in A-soil with and without OAM was roughly two orders lower than that in F-soil.

As speciation analysis in soil solutions revealed the presence of predominant inorganic As and minor organic As, such as MMA, DMA, and AsB (Supplementary Figs. S2 and S3), although the application of OAMs possibly enhances the methylation of As associated with microbial activities^37,38^. For F-soil, we separately measured As(III) and As(V) (Supplementary Fig. S2), and results showed that As(V) occupied considerable proportions in soil solution when the total dissolved As was low. In contrast, almost all (> 90%) dissolved As appeared as As(III) at higher dissolved As concentrations (> 10 μg kg^−1^), thus supporting previous observation^39^.

**Figure 2:**
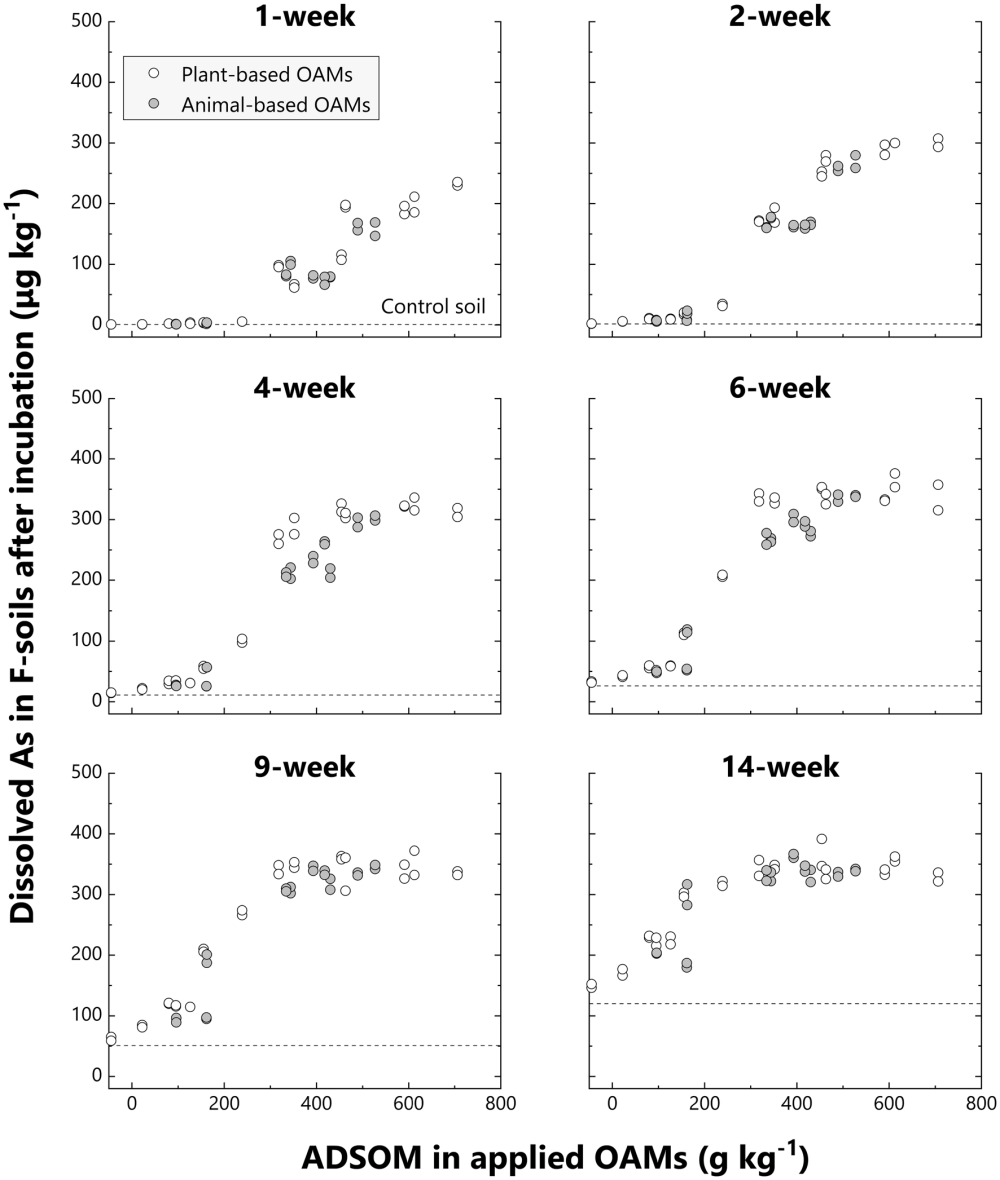
Relationship between dissolved As in F-soil after each incubation time and acid-detergent soluble organic matter (ADSOM) in the applied organic amendment (OAM). White circles and grey circles indicate plant-based OAMs and animal-based OAMs, respectively. The average of dissolved As in control soils is indicated by a dotted line in each incubation time for comparison.

**Figure 3:**
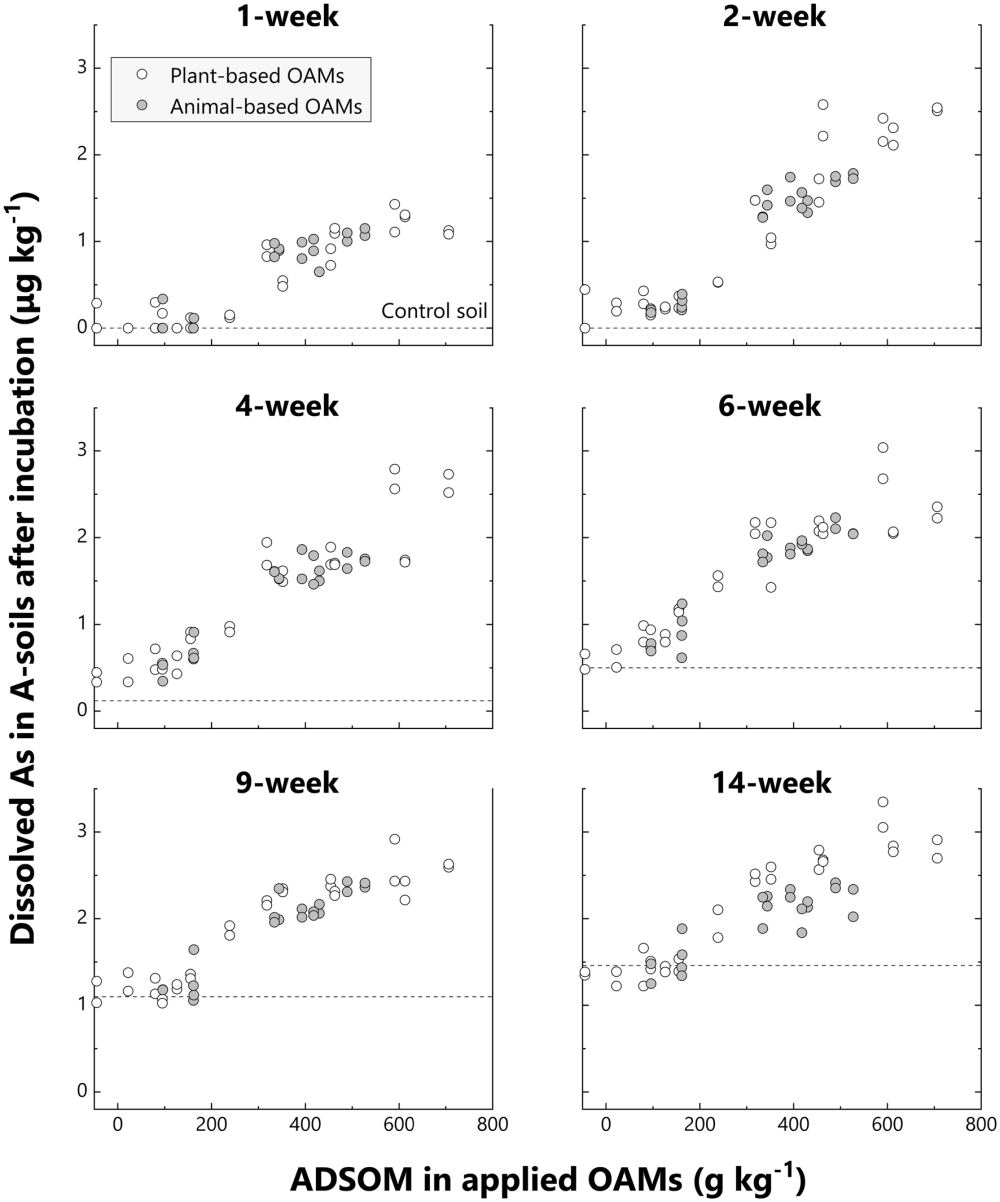
Relationship between dissolved As in A-soil after each incubation time and acid-detergent soluble organic matter (ADSOM) in the applied organic amendment (OAM). White circles and grey circles indicate plant-based OAMs and animal-based OAMs, respectively. Average of dissolved As in control soils is indicated by a dotted line in each incubation time for comparison.

Moreover, the amount of dissolved As in F-soil increased sigmoidally, rather than linearly, with ADSOM increase in applied OAMs (Fig. 2). The limited increase in dissolved As with the addition of ADSOM-poor OAMs (left parts of sigmoidal curves) reflected the insufficient decrease in soil redox potential for substantially increasing As dissolution (Supplementary Figs. S4 and S5). On the contrary, none or slight differences in dissolved As in soils with ADSOM-rich OAMs (right parts of sigmoidal curves) suggest the existence of maximum limits for As dissolution. Except for the 1-week incubation, dissolved As reached the plateau at around 350 μg kg^−1^ in F-soil (Fig. 2). A similar trend was also observed in A-soils, but much more ambiguously (Fig. 3, Supplementary Fig. S6).

Differences in dissolved As among soils mixed with ADSOM-poor OAMs were small after a short incubation period, and dissolved As among soils mixed with ADSOM-rich OAMs were also small after a long incubation, especially in F-soil (Figs. 2 and 3). These facts indicate that it would be misleading to compare As dissolution in soils induced by each OAM based on the result from a particular incubation period. Instead, time-cumulative concentrations of dissolved As during a long-term incubation should be used and therefore defined the ratio of the cumulative dissolved As in soils with each OAM to that of control soils as:

$$\mathrm{Ratio\, of\, cumulative\, dissolved\, As}\,=\,\frac{\mathrm{Cumulative\, dissolved\, As\, in\, soils\, with\, each\, OAM}}{\mathrm{Cumulative\, dissolved\, As\, in\, control\, soils}}$$

**Figure 4:**
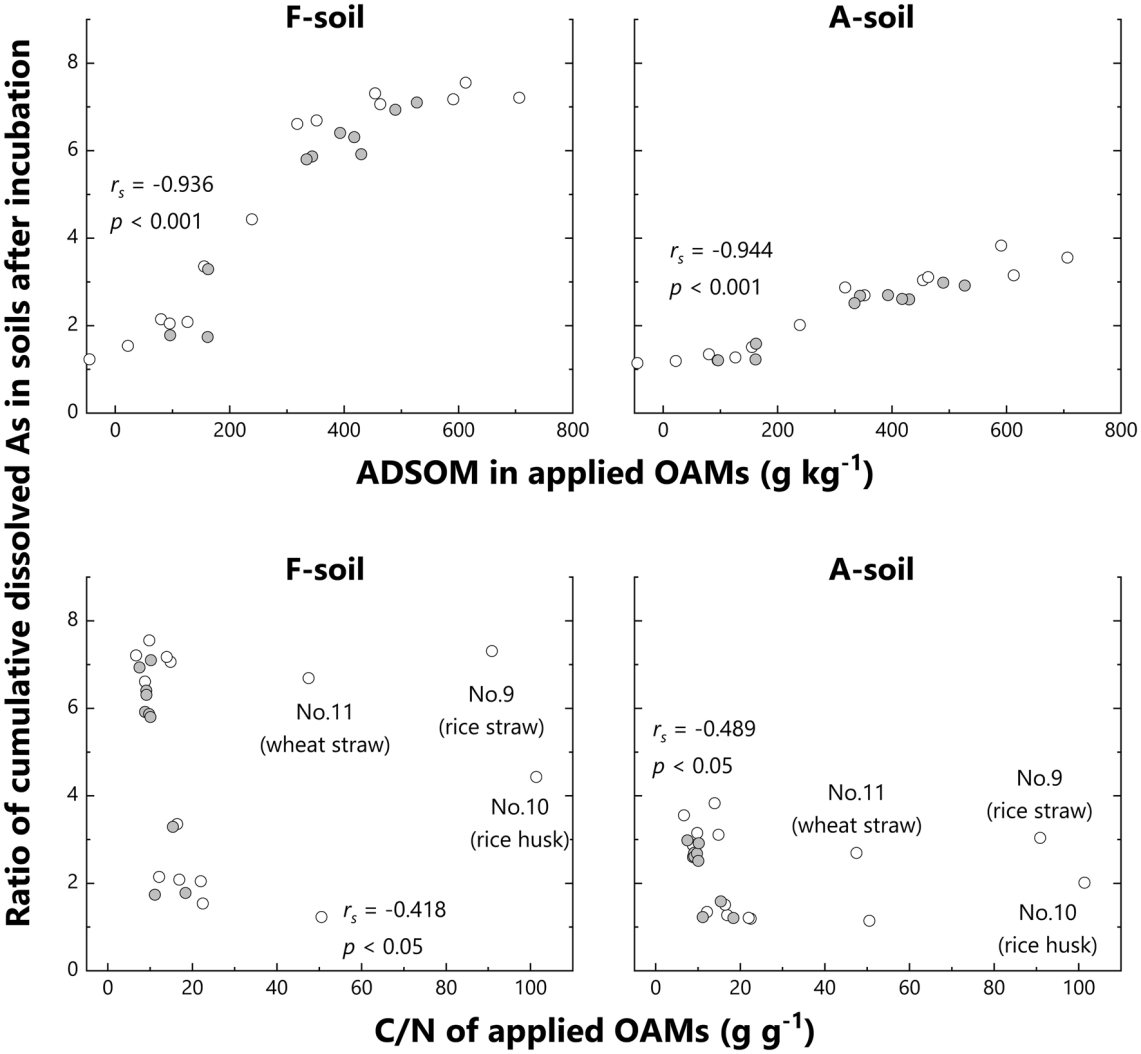
Relationship between the ratio of cumulative dissolved As in soils and acid-detergent soluble organic matter (ADSOM) contents, and carbon to nitrogen ratio (C/N) of organic amendments (OAMs) applied to the soils. The ratio of cumulative dissolved As is defined as the ratio of cumulative dissolved As in soils with each OAM obtained after the whole incubation period to that of control soils (i.e., soils without OAM). No. 9, No. 10, and No. 11 are soils with rice straws, rice husks, and wheat straws, respectively, which have abundant ADSOM and high C/N. Data are shown as the average of duplicates. *r*_*s*_ denotes Spearman’s rank correlation coefficient.

The ratio of cumulative dissolved As was strongly rank-correlated with ADSOM contents in applied OAMs (*r*_*s*_ > 0.936, *p* < 0.001 for both types of soils; Fig. 4). Notably, cumulative dissolved As in A-soil responded to the application of OAMs to a lesser extent compared to that in F-soil. According to the rough estimation from Fig. 4, cumulative dissolved As doubles when 0.5% wt of an OAM containing 100 g kg^−1^ of ADSOM is added to F-soil, and an OAM containing 250 g kg^−1^ ADSOM is added to A-soil. However, the ratios of cumulative dissolved As for F-soil and A-soil were highly rank-correlated (*r*_*s*_ = 0.977, *p* < 0.001) despite their contrasting properties and the difference in their sensitivity to OAM applications. These results corroborated that the ADSOM content of OAMs could be used as an indicator to select appropriate OAMs based on their potential to increase the ratio of As dissolution against OAM-free soils irrespective of their properties. Note that total As contents and soil properties such as mineralogy should be considered while evaluating the absolute values of dissolved As.

The C/N level is a possible indicator of the enhancement potential of OAMs for As dissolution in soils. Several studies have shown that the decomposition of natural organic matter or OAMs with low C/N was faster than that with high C/N ratio^40–42^. Solaiman et al.^28^ demonstrated that As dissolution from As-bearing Fe oxide-coated sand was controlled by the C/N of added OAMs. Our previous study showed strong rank-correlations among dissolved As in soils, soil Eh, and C/N of applied OAMs^25^. However, the study examined only well-composted or well-fermented OAMs. The present study showed similar negative relationships (*r*_*s*_ < − 418, *p* < 0.05 for both soils) between the ratio of cumulative dissolved As and C/N of applied OAMs in both F- and A-soil (Fig. 4). However, No. 9 (rice straw), 10 (rice husk), and 11 (wheat straw) had large ratios of cumulative dissolved As despite their high C/N values, showing that C/N is not useful to evaluate the enhancement potential of plant-based fresh OAMs (i.e., OAMs with both high C/N and abundant ADSOM). This is consistent with reports of several studies, which indicated that the main structural components, rather than C/N, regulate the decomposability of the OAMs^43,44^. ADSOM comprises easily to moderately decomposable components of OAMs, namely non-fiber organic substances and hemicellulose^30^; therefore, it would be a more widely applicable indicator than C/N. A drawback in using ADSOM is the complicated and time-consuming quantifying method, but an easy alternative using near-infrared spectroscopy has been suggested by Fujiwara et al.^32^.

### Mechanisms of additional As dissolution in soils caused by organic amendments

Anaerobic conditions promote As dissolution, as also observed in the present study (Supplementary Fig. S7), based on the following mechanisms: (1) the desorption of As(III) from soil solid phases following the reduction of As(V) to As(III), and (2) the release of As during the reductive dissolution of As-bearing Fe oxides^45^. In this study, we assumed that ADSOM corresponds to decomposable OMs during long-term anaerobic conditions. According to Mukai and Oyanagi^30^, ADSOM is mainly composed of non-fiber OMs and hemicellulose and closely agrees with OMs decomposable within 3 months in aerobic soils. Although the decomposition rate of the ADSOM fraction under anaerobic conditions is unknown, our study showed negative relationships between soil Eh and ADSOM in applied OAMs (Supplementary Figs. S5 and S6). Therefore, ADSOM enhanced microbial respiration and the subsequent development of reduced conditions.

**Figure 5:**
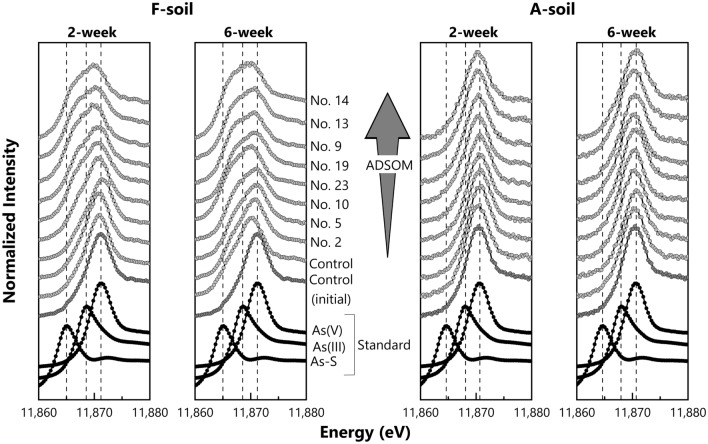
Arsenic K-edge X-ray absorption near edge structure spectra of solid phases in F-soils and A-soils, with or without selected organic amendments. The samples were collected after the 2- and 6-week incubations. Black plots denote the spectra of the reference materials, namely arsenate adsorbed on ferrihydrite [As(V)], sodium arsenite [As(III)], and orpiment (As-S). The spectrum of the initial control soil is shown as a dark gray line in all subfigures for comparison.

**Figure 6:**
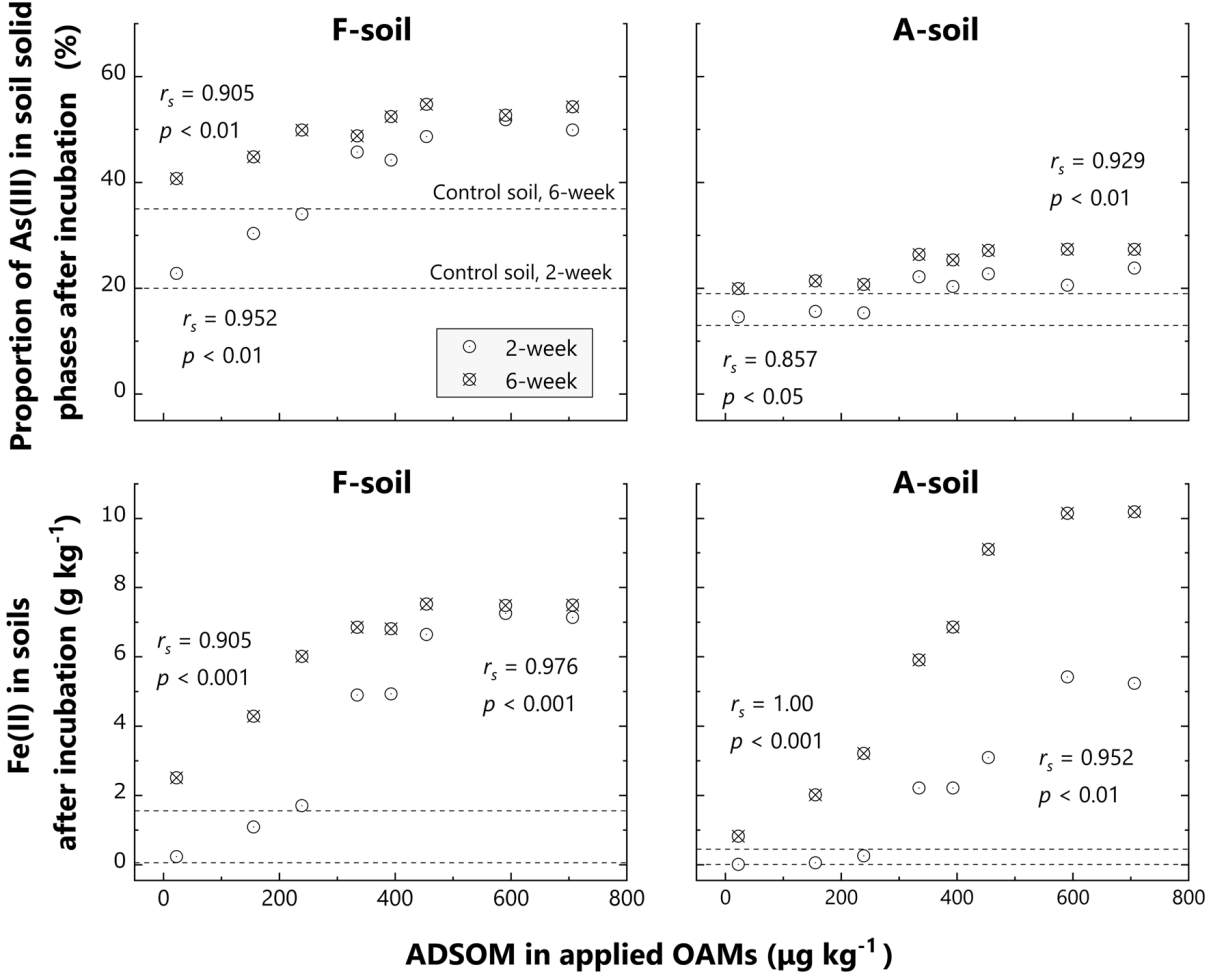
Relationship between acid-detergent soluble organic matter (ADSOM) in applied organic amendments (OAMs), proportion of As(III) in solid phases (upper layers) of soils, and Fe(II) contents in soils (lower layers) after 2- and 6-week incubation. The soil samples were with or without selected OAMs (2, 5, 9, 10, 13, 14, 19 and 23). The proportion of As(III) or Fe(II) content in control soils is indicated by a dotted line in each incubation time for comparison: the upper line is the 2-week incubation, and the lower is the 6-week incubation. Data are shown as the average of duplicates. *r*_*s*_ denotes Spearman’s rank correlation coefficient.

**Figure 7:**
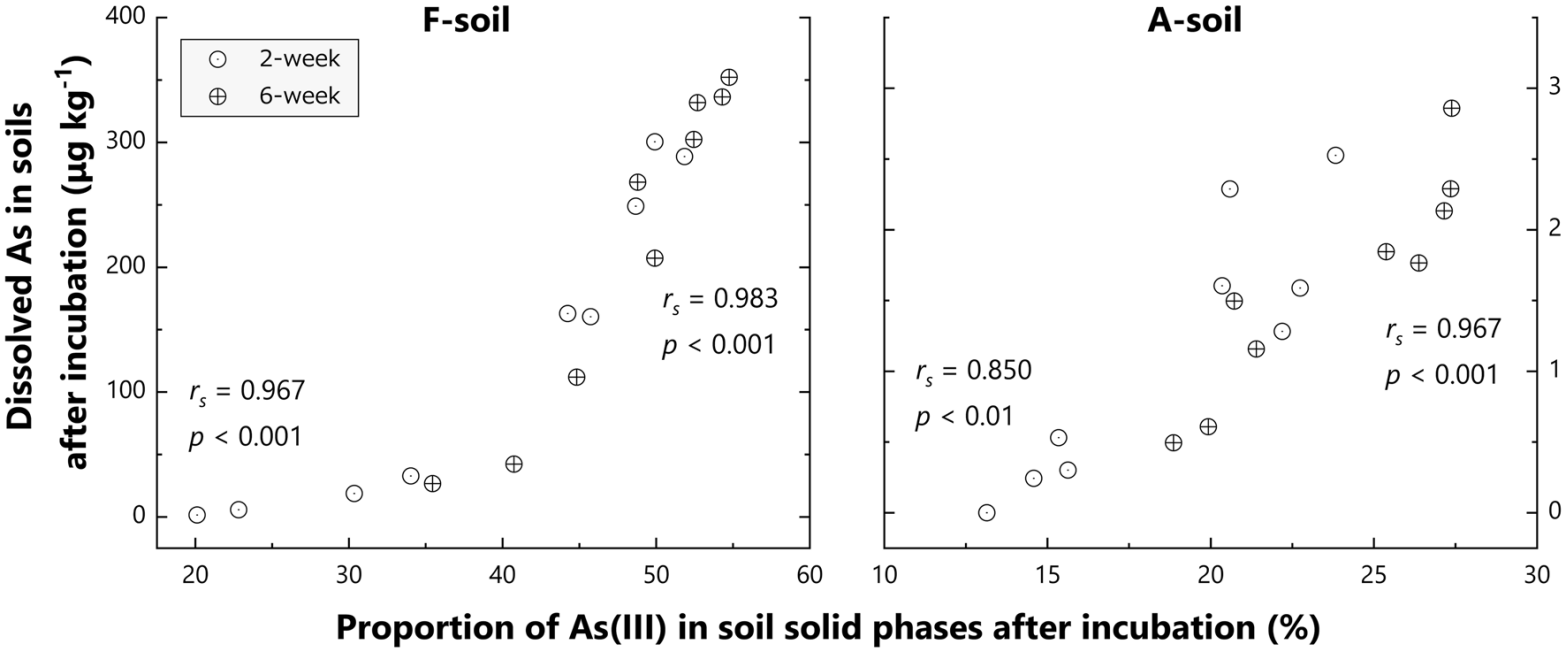
Relationship between dissolved As and As(III) proportion in solid phases of soils with or without selected organic amendments (2, 5, 9, 10, 13, 14, 19 and 23) after 2- and 6-week incubation. Dissolved As is shown as the average of duplicates. *r*_*s*_ denotes Spearman’s rank-correlation coefficient.

Under reduced conditions, As(V) reduction to As(III) by microorganisms occurs in soils^46^. The linear combination fitting of As K-edge XANES spectra demonstrated that As(III) increased from 19 to 55% for F-soil, and from 13 to 27% for A-soil with ADSOM increase in the applied OMAs (Figs. 5 and 6; *r*_*s*_ > 0.905, *p* < 0.01 for F-soil, *r*_*s*_ > 0.857, *p* < 0.05 for A-soil), as well as a decrease in soil redox potential (Supplementary Table S3). Moreover, there is a strong positive rank correlation between As(III) in solid phases and dissolved As in soils (Figs. 5 and 7; *r*_*s*_ > 0.967, *p* < 0.001 for F-soil, *r*_*s*_ > 0.850, *p* < 0.01 for A-soil), which is consistent with a previous study^13^ since the affinity of As(III) for soil solid phases is generally lower than that of As(V)^39,45^. Thus, ADSOM in applied OAMs was demonstrated to enhance As(V) reduction and subsequent As dissolution through its microbial decomposition during anaerobic conditions.

Our study also demonstrated that ADSOM in applied OAMs promoted the reduction of As-bearing Fe oxides: the contents of Fe(II) in soils after 2- and 6-week incubations increased as ADSOM contents of applied OAMs did (Fig. 6), and dissolved Fe showed a similar trend (Supplementary Figs. S8 and S9). Supplementary Figure S10 shows the positive relationships between dissolved As and Fe(II) and dissolved Fe, suggesting substantial contributions of the reductive dissolution of As-bearing Fe oxides to the increase in dissolved As in anaerobic soils, even if released As is resorbed onto newly formed secondary Fe phases, especially in the former stage of anaerobiosis^16^. Thus, our results showed that ADSOM in OAMs accelerated the reduction processes in soils and consequent As dissolution from the solid phases.

### Differences in As solubility between F-soil and A-soil

The amount of dissolved As in A-soil with and without OAMs was approximately two orders lower than that in F-soil, although pseudo-total As contents were comparable in both. This could be attributed to differences in the rate of As(V) reduction and amount of remaining Fe oxides during incubation between the two soils.

The As K-edge XANES analysis revealed that As(V) in A-soil was hardly reduced (Fig. 5). The LCF of XANES spectra indicated that the As(V) proportion in A-soil was 68–80% (initial: 87%) even after a 6-week anaerobic incubation, while that in F-soil was only 35–55% (initial: 78%) (Fig. 5, Supplementary Table S3); this could not be explained in terms of redox potentials as there is only a slight difference in redox potentials between the two soils (Supplementary Figs. S5, S6 and Table S3). It is possible that As(V) reduction was hindered by distinct micro-environments around As(V) in A-soil (Andosols); as Chevallier et al.^47^ suggested, allophane forms stable aggregates with a mesopore network (2–50 nm), which can prevent obstructed organic matter from enzyme and microbial action. Several other studies have implied the sequestration of Fe oxides by amorphous Al minerals^48,49^ and the low microbial reduction rate of amorphous Fe oxides in Andosols^50,51^. Thus, it is presumable that some parts of As(V) sorbed on Fe oxides and that other phases were to be sequestered into a stable aggregate. Consequently, the acceleration of As(V) reduction by the attachment of microbial cells to As-bearing phase^52^ would be prevented. This hypothesis is consistent with our previous observation that there is a strong negative correlation between As dissolution and the Al_o_ content of anaerobic soils ^51^. Therefore, incorporation of As(V) in stable aggregates could explain, at least partially, the lower reduction of As(V) observed in A-soil.

Finally, after 2-week and 6-week incubations, the ratio of dissolved As to As(III) in solid phases of A-soil was one order smaller than that for F-soil (Supplementary Fig. S11), implying that the solid phases in A-soil efficiently sorbed As(III) compared to those in F-soil, even under anaerobic conditions. This is partly because of the much larger amount of remaining Fe oxides—especially amorphous Fe oxides—, in A-soil than in F-soil. Additionally, the abundant amorphous Al minerals in A-soils would contribute to lowering As(III) dissolution to some extent, although they sorbed As(III) less effectively than Fe oxides^5,6^.

## Conclusion

This study tested the hypothesis that contents of ADSOM in OAMs can help estimate the potential of OAMs in accelerating As dissolution in applied soils, by subjecting two contrasting soil types, Andosol and Fluvisol, to anaerobic incubation. Our findings can be summarized as follows:

1. Acid-detergent soluble organic matter, mainly composed of non-fiber organic matter and hemicellulose, in OAMs can be an accurate and practical indicator of the enhancement potential of OAMs for As dissolution in anaerobic soils.
2. The reduction of As(V) and Fe oxides in soils tended to increase with higher ADSOM contents in applied OAMs, indicating that ADSOM decomposition and the subsequent development of reducing soil conditions are the main causes for the increase in dissolved As in soils with OAMs.
3. Dissolved As in Andosol was roughly two orders lower than that in Fluvisol, and less sensitive to the application of OAMs, possibly due to the limited As(V) reduction and abundant Fe oxides that remained in Andosol even after prolonged anaerobic incubation.

Ultimately, this study, being the first to do so, analyzed the relationship between ADSOM in OAMs and As dissolution from anaerobic soils with added OAMs. Our findings can aid in the selection of appropriate OAMs to be applied to paddy fields, in order to minimize As exposure to rice plants, and in the development of better soil management practices.

## Supplementary Information

Supplementary Information 1.

Supplementary Information 2.

## Data Availability

All data are included in this published article and its Supplementary Information files.
