# Supplementary material for: Enhanced dissolution of arsenic in anaerobic soils upon organic amendment application: acid detergent-soluble organic matter as a potential indicator
Source: Sci Rep. 2023 Jan 5;13:217. doi: 10.1038/s41598-022-27325-1 (PMC9816317; doi:10.1038/s41598-022-27325-1)
Supplement: Supplementary file 2 — Supplementary Information 2. [file 41598_2022_27325_MOESM2_ESM.pdf]

Enhanced dissolution of arsenic in anaerobic soils upon organic amendment application: Acid detergent-soluble organic matter as a potential indicator

Aomi Suda[1,*], Koji Baba[2], Gen Sakurai[1], Manami Furuya[2], and Noriko Yamaguchi[1]

[1] Institute for Agro-Environmental Sciences, NARO, Kannondai 3-1-3, Tsukuba, Ibaraki 305-8604, Japan
[2] Research Center for Advanced Analysis, NARO, Kannondai, Tukuba, Ibaraki 305-8604, Japan
*suda_aomi@affrc.go.jp

Analysis of soil samples

Soil pH was measured in the water using a pH meter with a glass electrode with an air-dried soil-to-water ratio of 1:2.5 (w/v). Soil organic matter (SOM) content was estimated using the following equation: 1.72 × organic carbon. It was calculated using an NC analyzer (Sumigraph NC-900; Shimadzu, Kyoto, Japan). Dithionite-citrate-, acid oxalate-, and pyrophosphate-extractable elements were extracted as described by Blakemore et al.(1987). The extraction of pseudo-total As was performed by digesting an aliquot of ground soil sample with a mixture of nitric acid and perchloric acid on a hot plate, and the elements in the extracts were quantified using inductively coupled plasma-optical emission spectrometry (Agilent 700 Series, Agilent Technologies, Santa Clara, CA, USA) or inductively coupled plasma mass spectrometry (ICP-MS; NexION300XX, PerkinElmer, Waltham, MA, USA) after filtration and dilution. The contents of clay-, silt-, and sand-size particles were quantified using a pipette method, following SOM digestion, with hydrogen peroxide. For A-soil, clay particles could not be dispersed by adding diluted sodium hydroxide, hydrochloric acid, or sodium hexametaphosphate. Therefore, clay content quantification involved subtracting silt, sand, and SOM weights from the entire soil weight. Soil silt and sand content were determined using the pipette method after removing inhibitors against clay dispersing (i.e., Fe oxides and amorphous Al minerals) using Jeffries method (Wada and Kawano 1987).

References

Blakemore *et al. Methods for Chemical Analysis of Soils*. 71-76 (NZ Soil Bureau, 1987).
Wada, K. & Kawano, T. Use of Jeffries acid oxalate treatment in particle-size analyses of Ando soils. *Geoderma* **20**, 215-224 (1978).

Analysis of dissolved As and Fe in soils

As speciation in the soil solution was determined using high-performance liquid chromatography (HPLC)-ICP-MS (HPLC: PerkinElmer Flexar HPLC System) within 48 hours after soil solution sampling. The injection volume of the soil solution was 10 μL for F-soil and 50 μL for A-soil. The measured standard As species were arsenite [As(III)], arsenate [As(V)], monomethylarsonic acid (MMA), dimethylarsinic acid (DMA), and arsenobetaine (AsB). In a few cases, unidentified species in small amounts were detected in soil solutions and were quantified using the standard curve for As(V). The limit of detection (LOD) and the limit of quantification (LOQ) of As concentration in soil were 3 σ and 10 σ, respectively, in 10 method blanks spiked with analytes. The highest values of LOD and LOQ among the standard were applied uniformly to all species: LOD was 0.28 and 0.11 μg kg^{-1}, and LOQ was 0.94 and 0.37 μg kg^{-1} for F-soil and A-soil, respectively. The total amount of dissolved As in soil solutions was calculated by adding the values higher than the LOD for all species, including those unidentified. When none of the species exceeded the LOD, the total As of the soils was fixed at 0 μg kg^{-1} in the data analysis. The concentration of dissolved Fe was measured using ICP-OES. The LOD and the LOQ of Fe concentrations were 3 σ and 10 σ, respectively, in 10 method blanks. LOD was 0.011 mg kg^{-1} and LOQ was 0.036 mg kg^{-1}. For each dissolved As species and dissolved Fe, data lower than the LOD were reported as 0 mg kg^{-1}, and those above the LOD but below the LOQ were reported as the calculated values .

Analysis of ferrous iron in soils

To quantify Fe(II) in incubated soils, the control soils and soils with OAMs (2, 5, 9, 10, 13,14, 19, and 23) were collected after 2- and 6-week incubation. The extraction and measurement of Fe(II) in soils were performed according to Kumada and Asami (1957) with slight modifications. The incubated soil was rapidly mixed with 220 mL of 1.14 mol L^{-1} sodium acetate solution (pH 2.8, adjusted by hydrochloric acid) in a 250-mL polyethylene bottle. The bottle stood for 2 h with occasional shaking. Approximately 20 mL of the suspension was transferred to a 50-mL polyethylene centrifuge tube and then centrifuged at 1700 x *g* (3500 rpm) for 5 min. The supernatant was filtered with a 0.2-μm membrane filter, and a certain amount of filtrate was immediately added to the mixture of 0.2% α, α'-dipyridyl solution, acetate buffer (pH 5), and ultrapure water. The concentration of Fe(II) in the mixture was determined by an ultraviolet-visible spectrophotometer (Evolution 200, Thermo Fisher Science, MA, USA) at a wavelength of 522 nm.

References

Kumada, K. & Asami, T. A new method for determining ferrous iron in paddy soils. *Soil Sci. Plant Nutr*. **3**, 187–193 (1957).

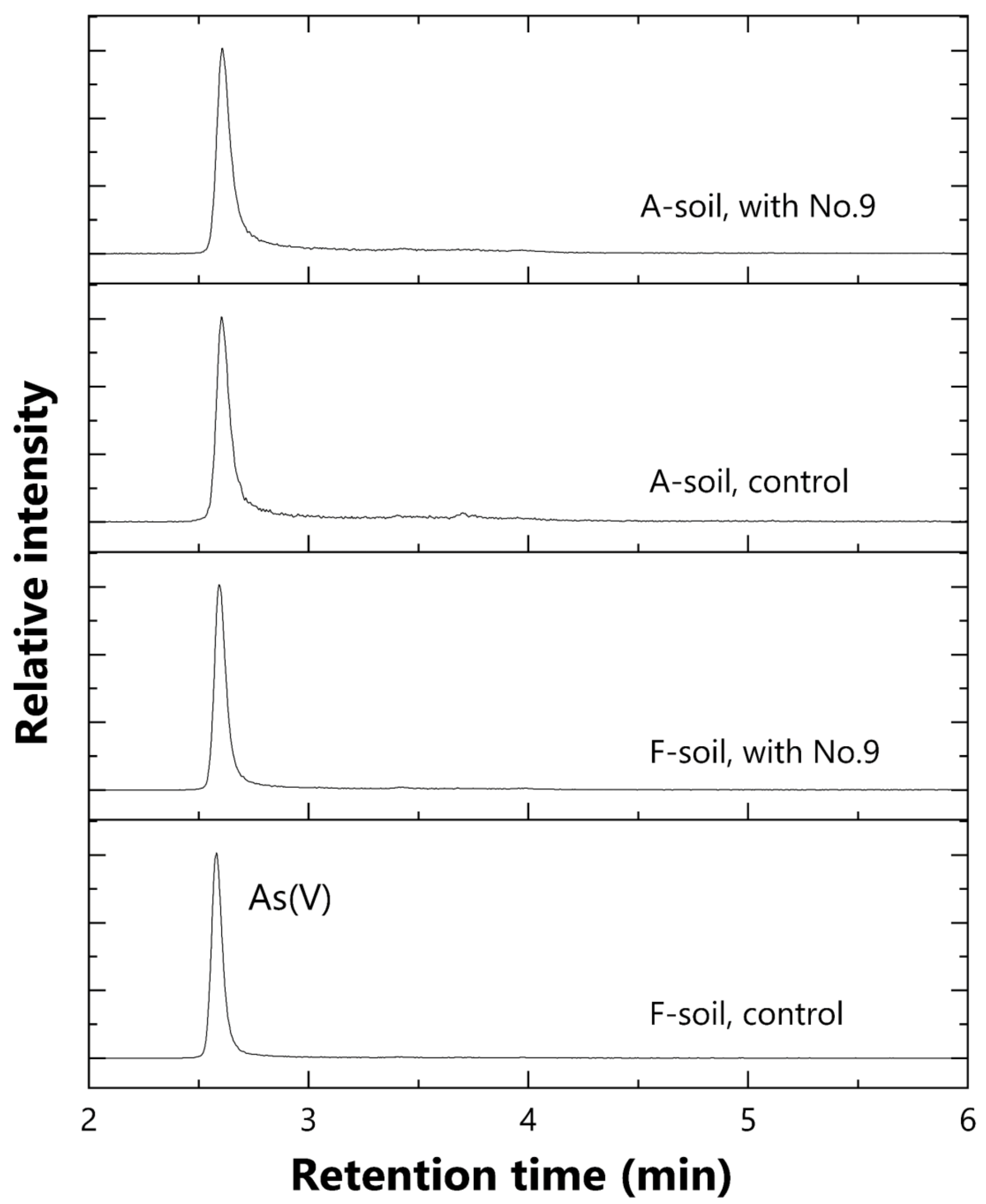

Figure S1. Chromatograms showing the presence of arsenic (As) species in the extracts of control soils and No. 9 (rice straw)-applied soils after a 6-week incubation. Soil pastes after XANES measurement were air-dried and then extracted using concentrated nitric acid heated for 4 h of heating at 100 ℃, following the method described by Baba et al. (2008). Arsenite remained undetected because it was oxidized to arsenate (As[V]) during nitric acid extraction. No significant peaks of organic As species, namely monomethylarsonic acid (at 3.4 min), dimethylarsinic acid (at 4 min), and arsenobetaine (at 5.6 min), were observed despite these methylated species being considered stable during extraction, implying that these organic As species occupied no or negligible proportions of total As in the examined soils.

Baba, K. *et al.* Arsenic speciation in rice and soil containing related compounds of chemical warfare agents. *Analytical Chemistry*, **80**, 5768-5775 (2008).

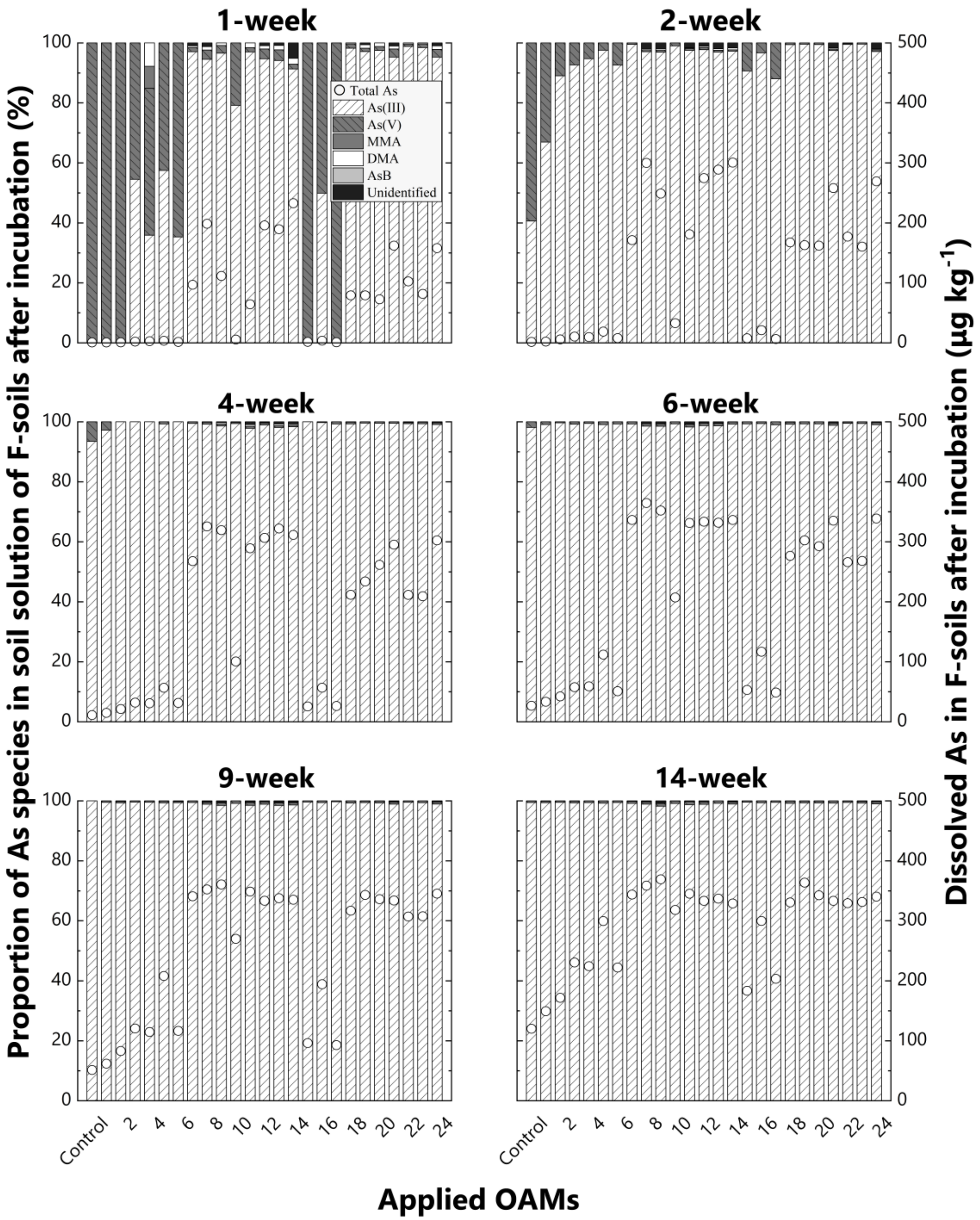

Figure S2. Graph depicting the proportions of arsenic (As) species in F-soil solution after each incubation period. Bars indicate the proportions of As species, and plots indicate total dissolved As in soils. Data are shown as the average of values from duplicate experiments.

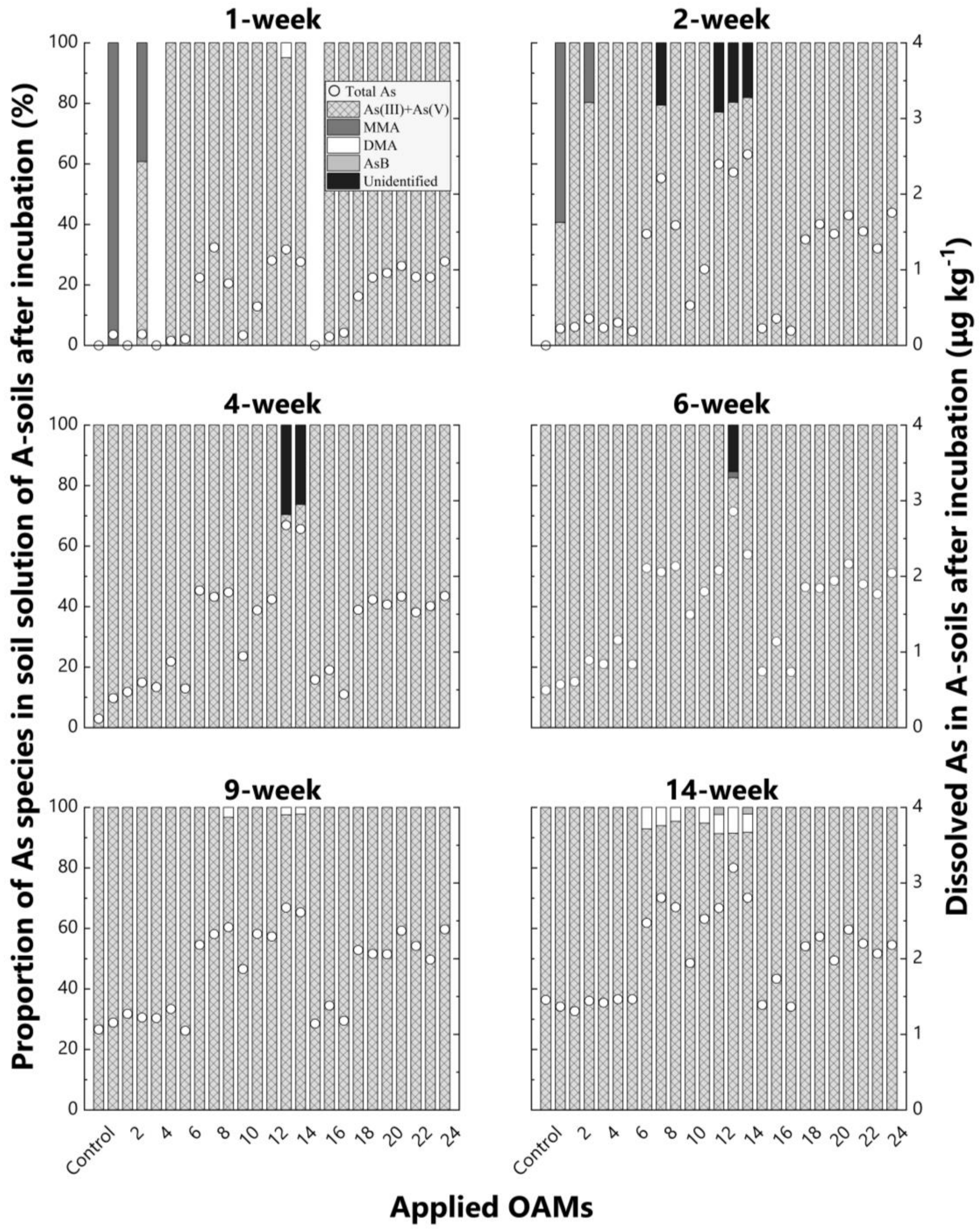

Figure S3. Graph depicting the proportions of As species in A-soil solution after each incubation period. Bars indicate the proportions of As species, and plots indicate total dissolved As in soils. Data are shown as the average of values from duplicate experiments. Exceptionally, only one sample was used for control soils and soils with No. 1 after a 6-week incubation because unexpected but apparent As(III) oxidation was observed in the other. Bars indicate the proportions of As species, and plots indicate total dissolved As in soils.

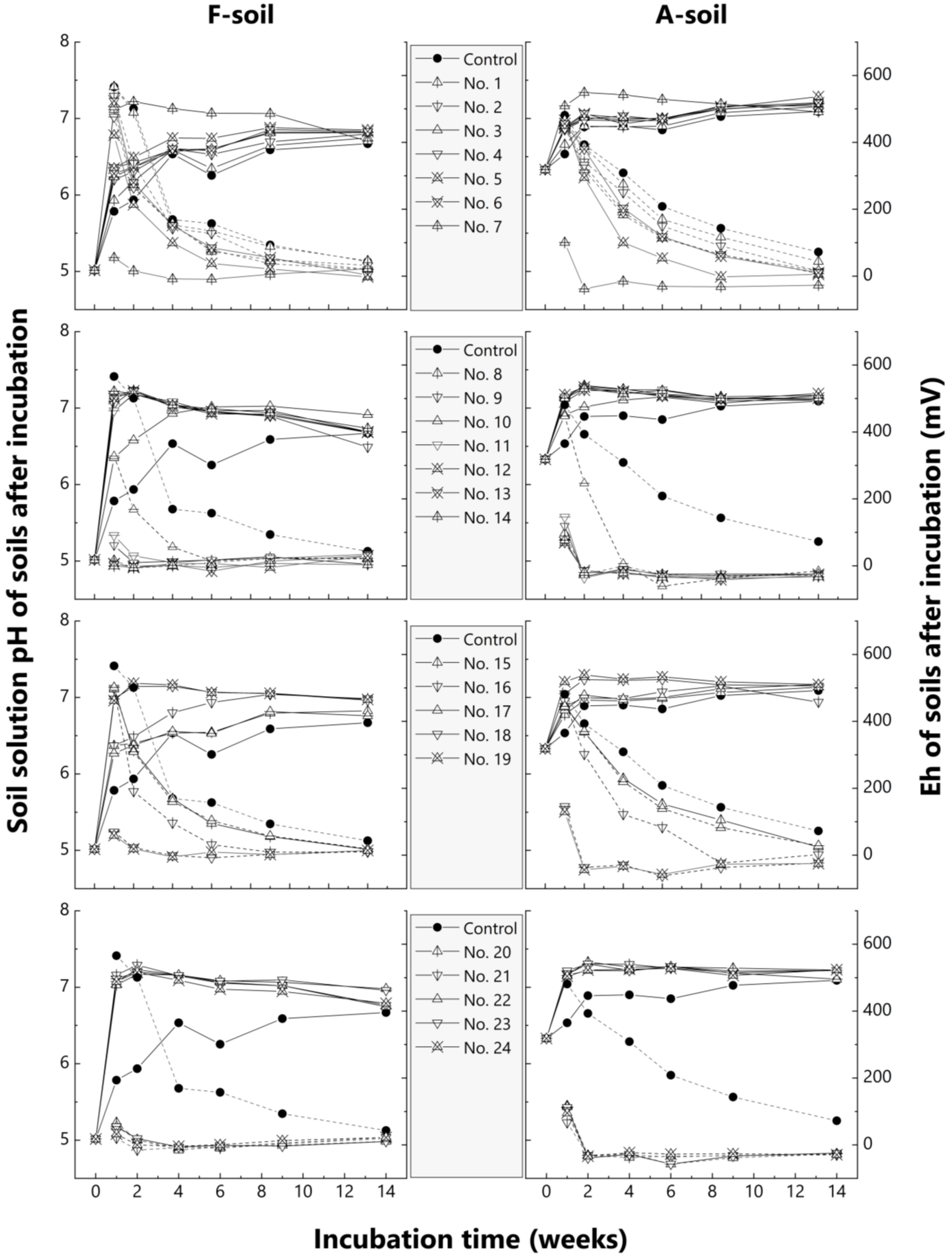

Figure S4. Time-course changes in pH and Eh of F-soils and A-soils during anaerobic incubation. Left panels correspond to F-soils and the ones on the right to A-soils. Control is shown in all panels for comparison. Data are shown as the average.

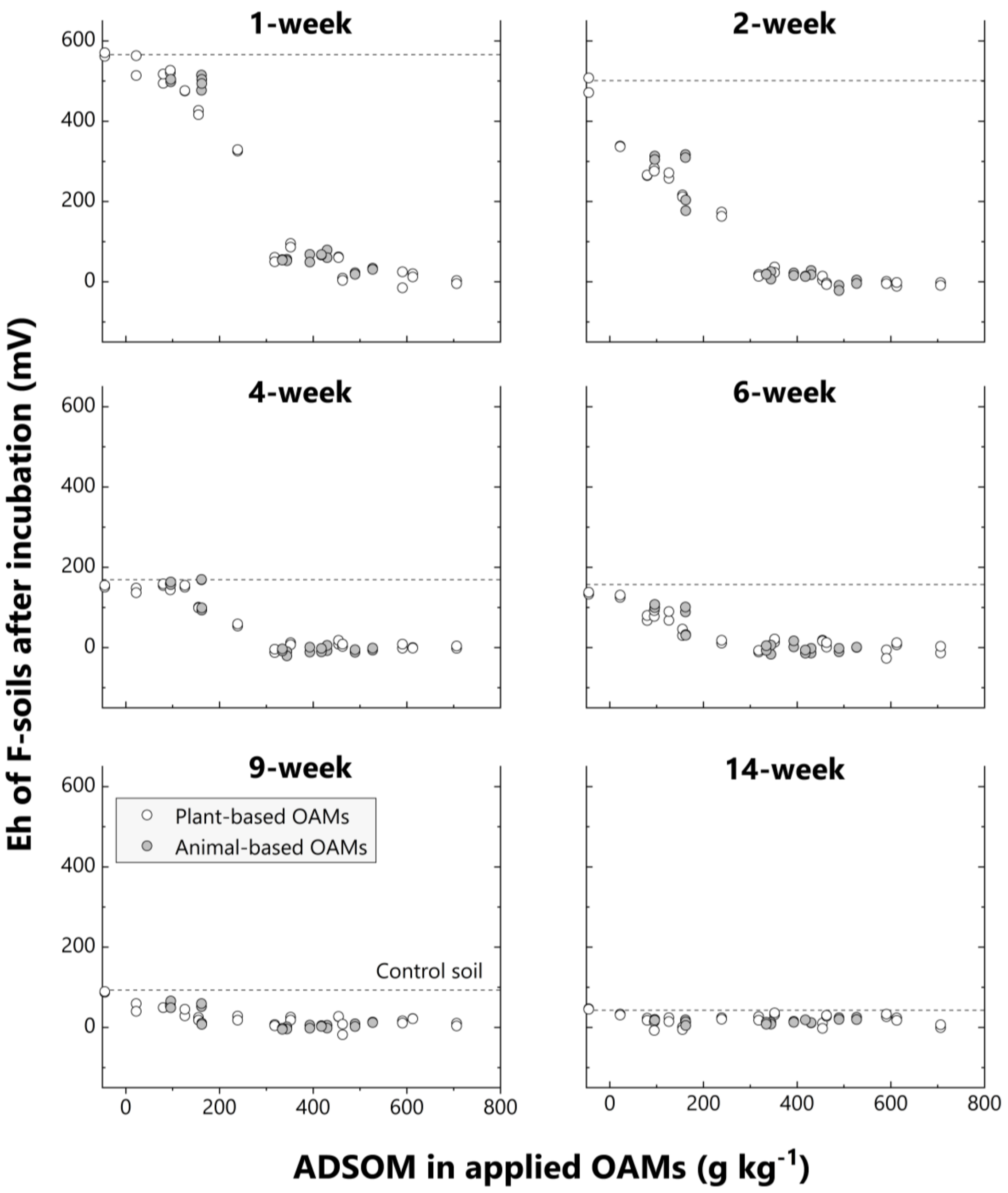

Figure S5. Relationships between the Eh of F-soils at each sampling time and acid-detergent soluble organic matter (ADSOM) in applied organic amendment (OAM). White and grey circles indicate plant-based and animal-based OAMs, respectively. The Eh of control soils is indicated as a broken line at each incubation time.

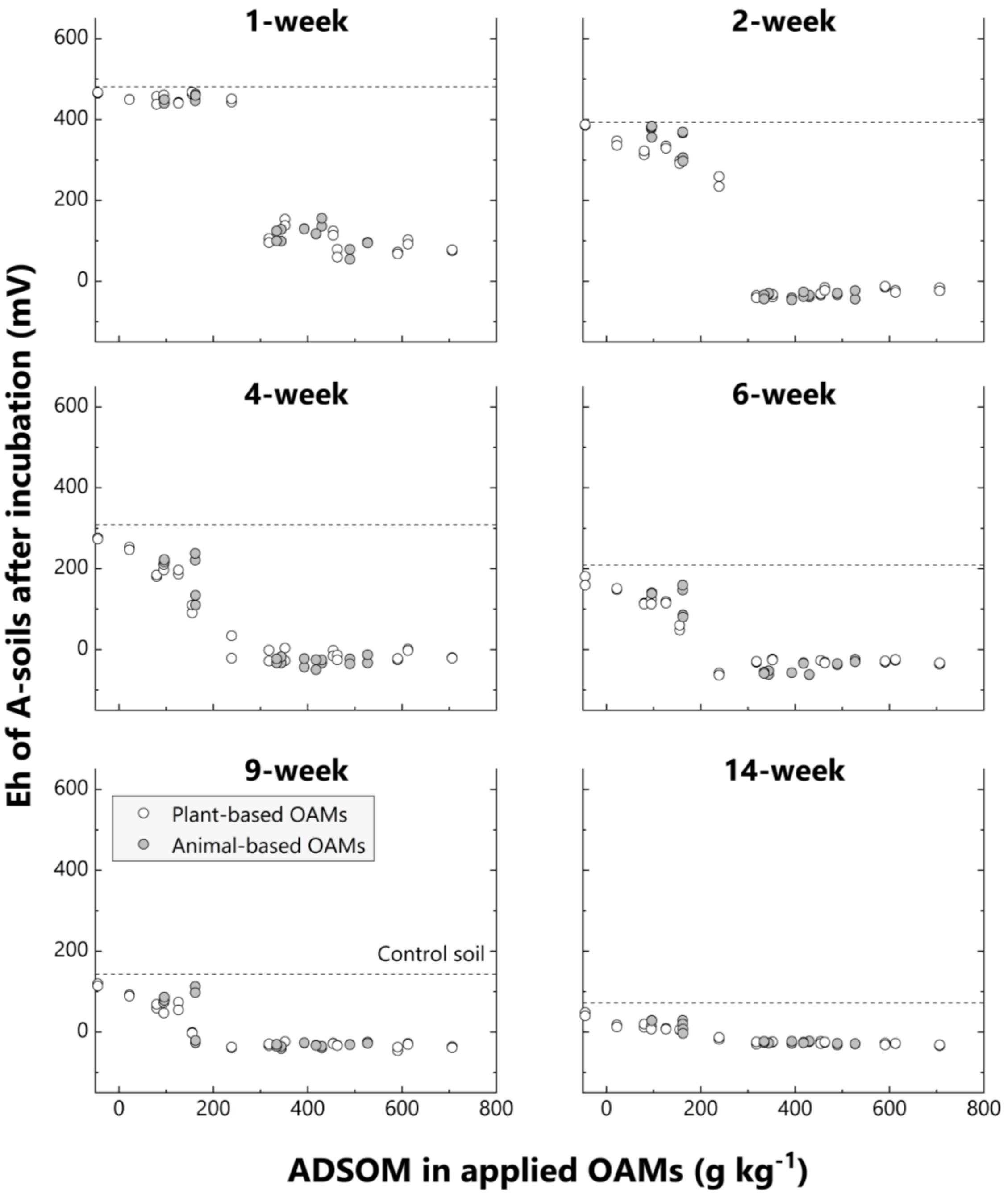

Figure S6. Relationships between the Eh of A-soils at each sampling time and acid-detergent soluble organic matter (ADSOM) in applied organic amendment (OAM). White and grey circles indicate plant-based and animal-based OAMs, respectively. The Eh of control soils is indicated as a broken line at each incubation time.

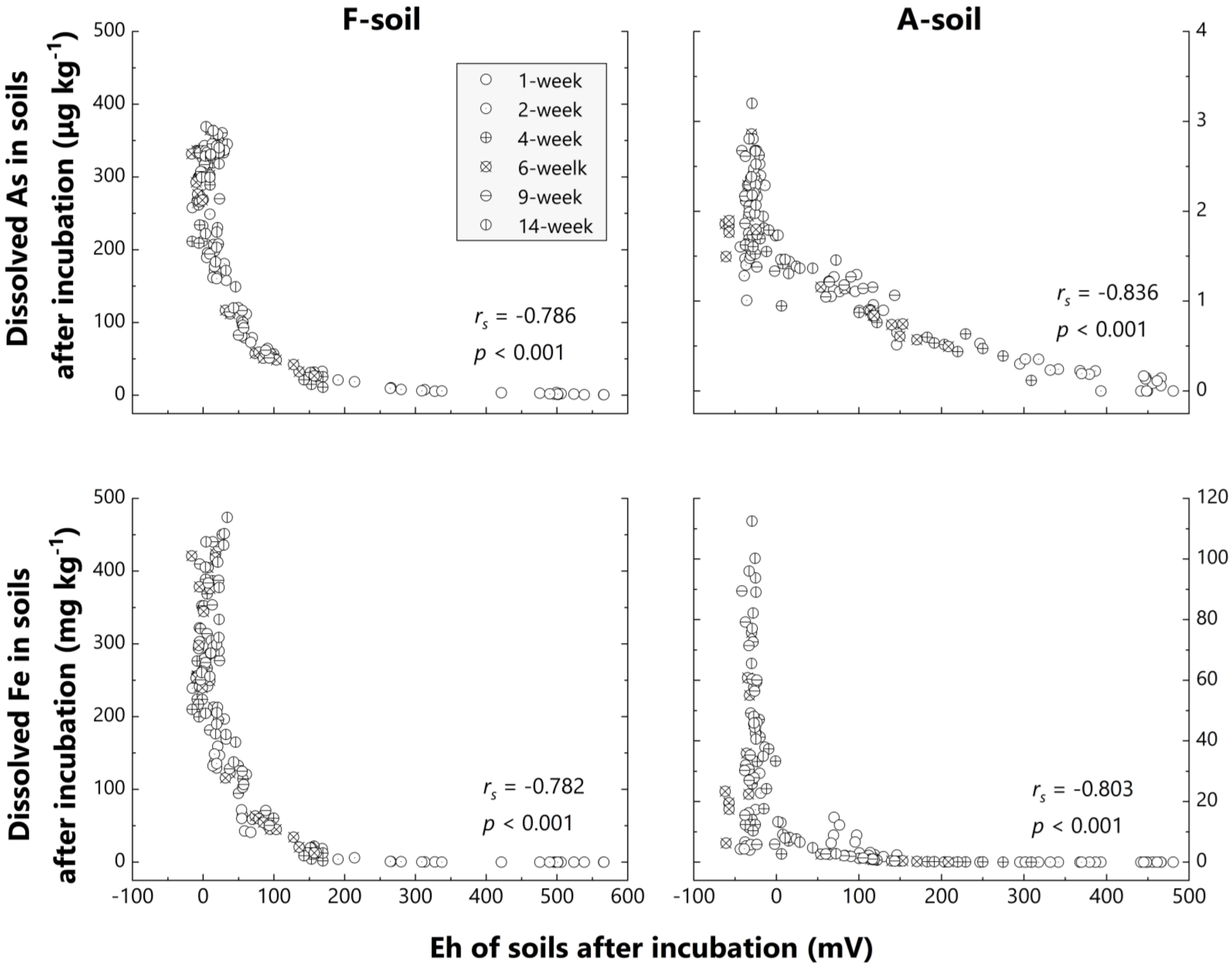

Figure S7. Relationships between dissolved As and soil Eh (upper panels) and dissolved Fe and soil Eh (lower panels) in soils. Data are shown as the average. r_s denotes Spearman's rank-correlation coefficient.

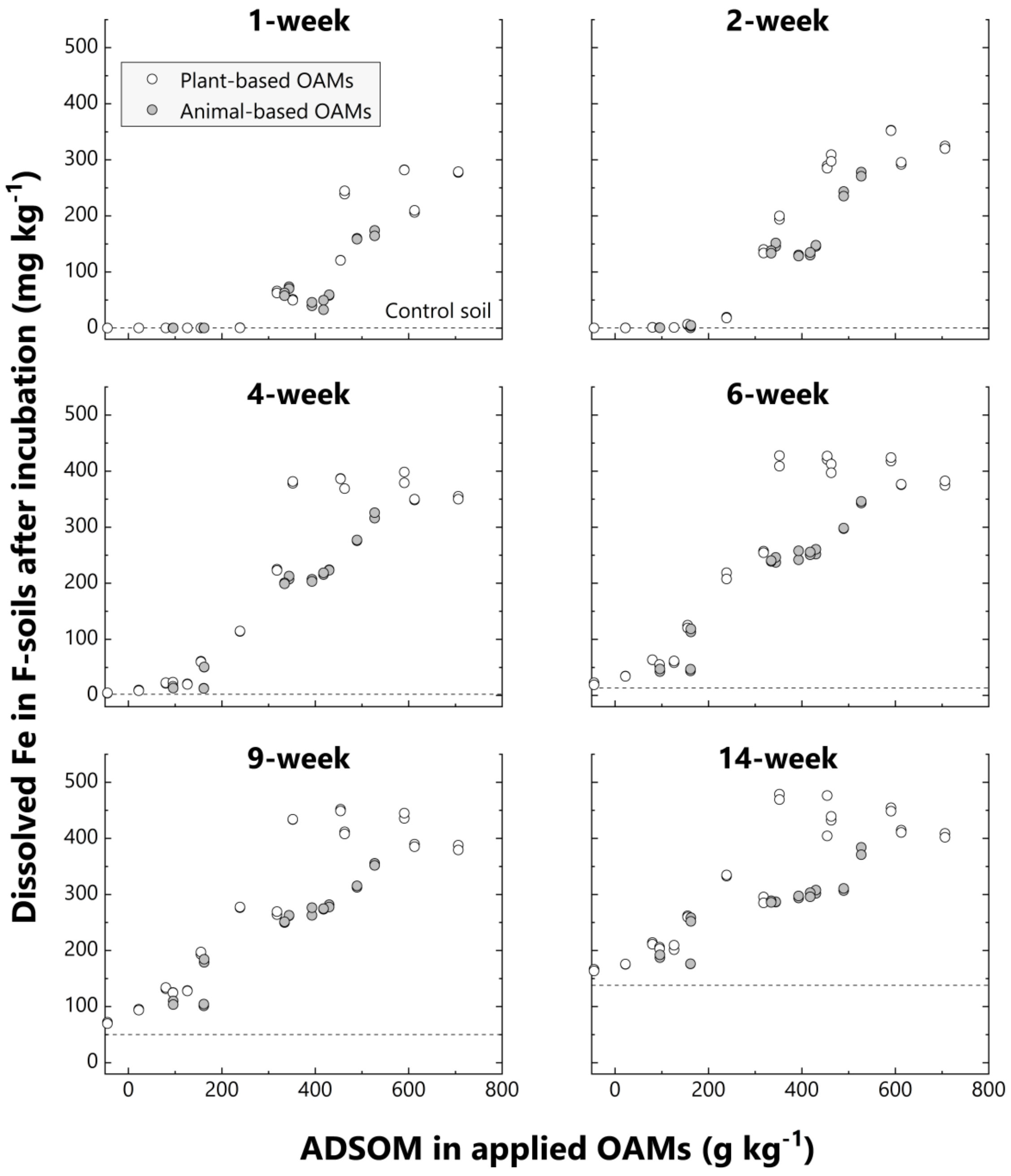

Figure S8. Relationships between dissolved iron (Fe) in F-soils after each incubation time and acid-detergent soluble organic matter (ADSOM) in applied organic amendment (OAM). White and grey circles indicate plant-based and animal-based OAMs, respectively. Dissolved Fe in control soils is indicated as a broken line at each incubation time.

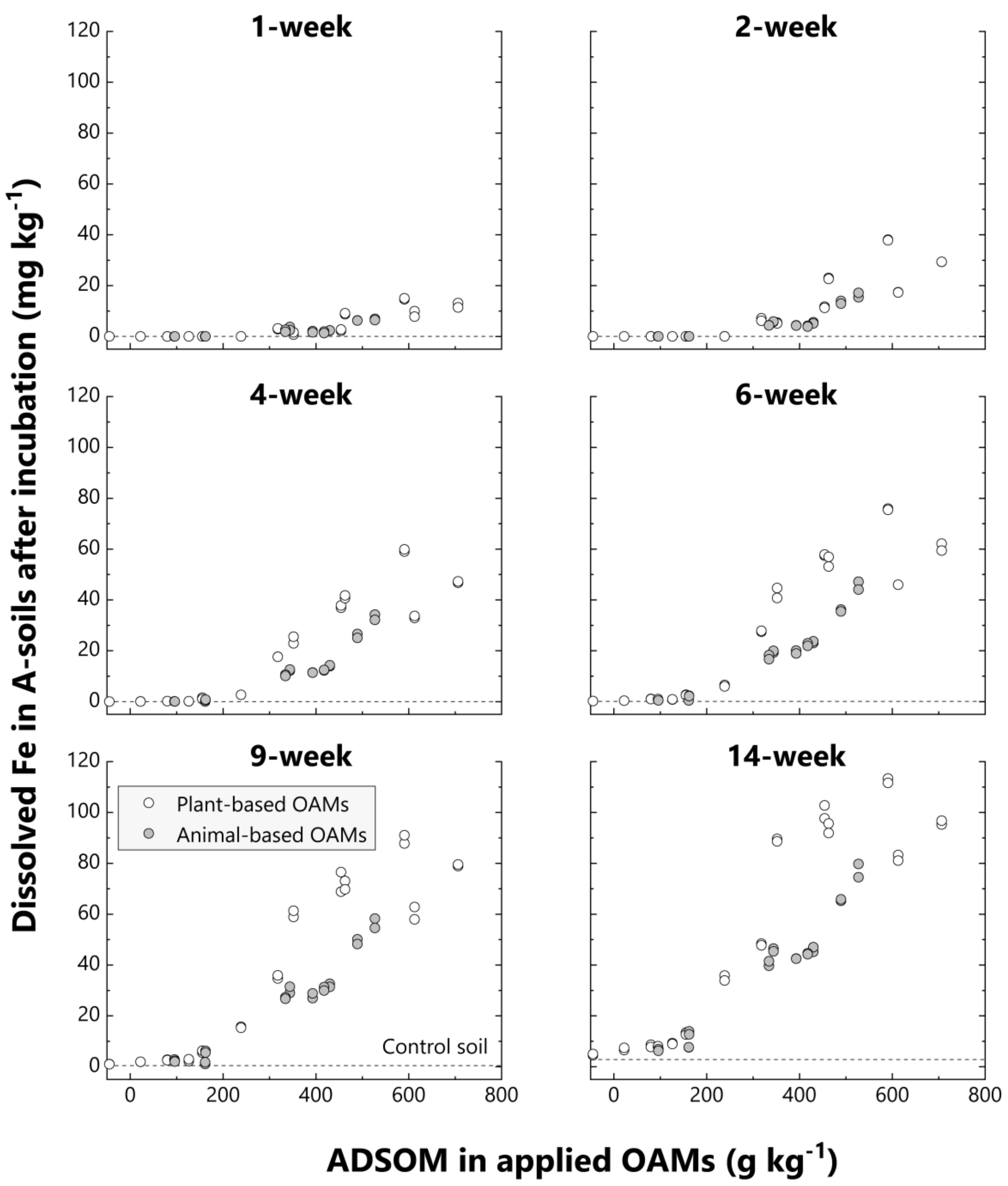

Figure S9. Relationships between dissolved iron (Fe) in A-soils after each incubation time and acid-detergent soluble organic matter (ADSOM) in applied organic amendment (OAM). White and grey circles indicate plant-based and animal-based OAMs, respectively. Dissolved Fe in control soils is indicated as a broken line at each incubation time.

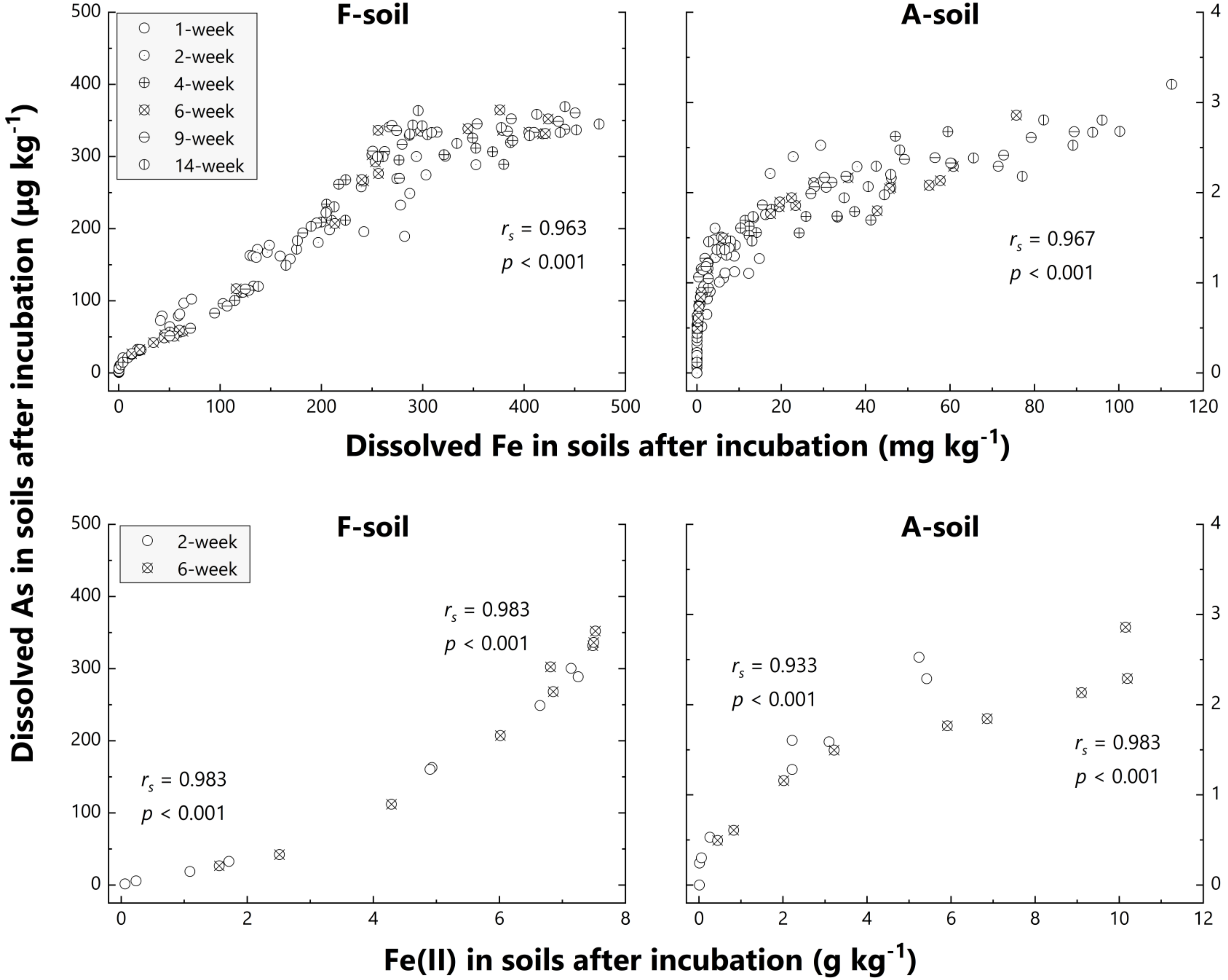

Figure S10. Relationships between dissolved As and dissolved Fe in all soil samples after all incubation times (upper panels); relationships between dissolve As and Fe(II) in soils with selected organic amendments after 2- and 6-week incubation (lower panels). Data are shown as the average of values from duplicate experiments. r_s denotes Spearman's rank-correlation coefficient.

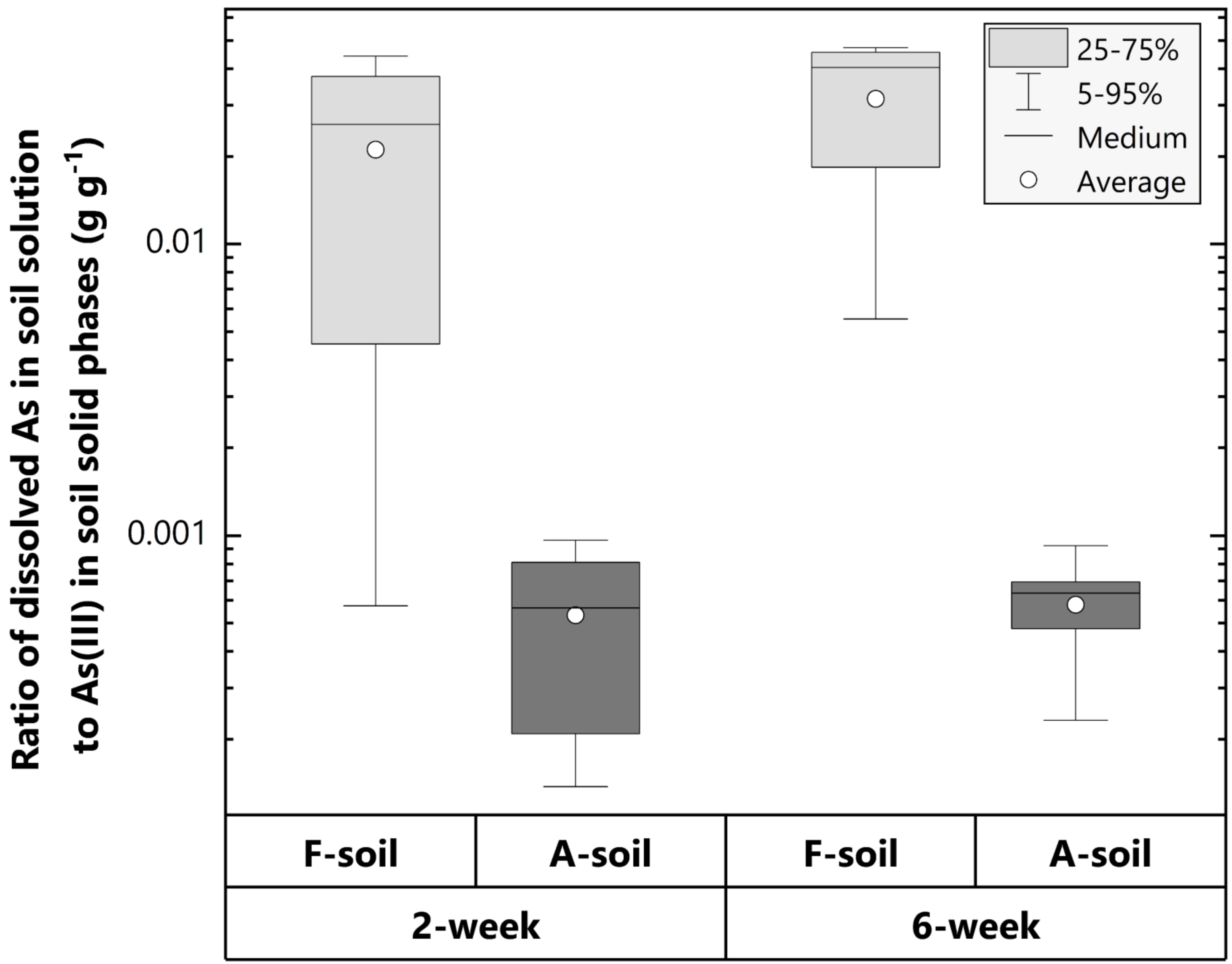

Figure S11. Ratio of dissolved As in soil solution to As(III) in solid phases of soils after 2- and 6-week incubation. Shaded boxes, lines, open circles, and whiskers indicate interquartile range, median, average, and 5 and 95 percentiles, respectively.

Table S1 Properties of the examined soils

Property	F-soil	A-soil
Sampling point (latitude, longitude)	36° 1'27.696", 140° 6' 28.836"	36° 1' 27.6594", 140° 6' 23.004"
Sampling depth	0-10 cm	0-10 cm
pH	5.72	7.14
Soil organic matter (g kg^{-1})	34.8	67.1
Oxalate-extractable Fe (g kg^{-1})	8.27	21.6
Dithionite-citrate-extractable Fe (g kg^{-1})	24.2	65.6
Oxalate-extractable Al (g kg^{-1})	3.82	68.3
Oxalate-extractable Si (g kg^{-1})	1.34	36.4
Pyrophosphate-extractable Al (g kg^{-1})	0.65	2.41
Pseudo-total As (mg kg^{-1})	13.6	11.3
Clay (g kg^{-1})	282	546
Silt (g kg^{-1})	272	198
Sand (g kg^{-1})	427	204
Soil texture	Light clay	Heavy clay
Soil order (WRB 2014)	Fluvisol	Andisol

References

IUSS Working Group WRB. (2014). World reference base for soil resources 2014. International soil classification. In *World Soil Resources Reports No.106*.

Table S2 Pseudo-total contents of C, N, Fe, P, and As in organic amendments (OAMs)

ID	Pseudo-total contents (mg kg^{-1} for As, g kg^{-1} for others)				
	C	N	Fe	P	As
—Plant-based OAM—					
No. 1	442	8.7	6.8	0.1	1.37
No. 2	352	15.7	11.5	0.3	2.30
No. 3	237	19.5	5.7	5.6	3.11
No. 4	374	22.1	5.3	7.3	1.45
No. 5	475	29.0	0.5	10.3	1.74
No. 6	373	16.9	13.3	0.8	1.93
No. 7	332	37.7	0.2	43.5	1.12
No. 8	449	45.7	0.0	22.2	0.65
No. 9	401	4.4	0.1	0.2	1.88
No. 10	419	4.1	0.0	0.1	0.37
No. 11	454	9.6	0.1	0.1	0.07
No. 12	472	31.8	0.3	0.4	0.27
No. 13	452	32.4	0.1	3.5	0.11
No. 14	507	75.7	0.0	11.1	0.04
—Animal-based OAM—					
No. 15	305	27.3	6.9	12.9	2.32
No. 16	409	26.5	3.8	13.9	1.80
No. 17	371	20.2	15.7	2.7	3.04
No. 18	366	41.5	0.4	21.3	0.97
No. 19	394	43.3	4.8	33.0	1.78
No. 20	403	44.1	4.4	32.6	0.97
No. 21	346	46.2	0.1	16.5	0.50
No. 22	314	32.1	0.1	23.0	0.59
No. 23	278	27.6	0.2	21.8	0.72
No. 24	435	42.7	0.1	11.9	0.14

Table S3 As species in soil solid phases estimated via linear combination fitting of X-ray absorption near edge structure analysis

Treatments	ADSOM	F-soil								A-soil							
		Eh	As(III)		As(V)		As-S		R-factor	Eh	As(III)		As(V)		As-S		R-factor
	($g\ kg^{-1}$)	(mV)	(%)	($mg\ kg^{-1}$)	(%)	($mg\ kg^{-1}$)	(%)	($mg\ kg^{-1}$)		(mV)	(%)	($mg\ kg^{-1}$)	(%)	($mg\ kg^{-1}$)	(%)	($mg\ kg^{-1}$)	
**— Initial —**																	
Control	-	-	19	2.5	78	11	3.1	0.4	0.004	-	13	1.4	87	9.9	0.0	0.0	0.002
**— After 2-week incubation —**																	
Control	-	501	20	2.7	78	11	2.1	0.3	0.005	393	13	1.5	86	9.7	0.9	0.1	0.003
No. 2	22	338	23	3.1	74	10	3.0	0.4	0.006	342	15	1.6	85	9.7	0.0	0.0	0.002
No. 5	155	214	30	4.1	64	8.7	6.0	0.8	0.008	295	16	1.8	84	9.5	0.0	0.0	0.003
No. 9	454	9	49	6.6	42	5.7	10	1.3	0.017	-32	23	2.6	74	8.4	3.0	0.3	0.003
No. 10	239	168	34	4.6	59	8.0	7.1	1.0	0.009	247	15	1.7	83	9.4	1.4	0.2	0.003
No. 13	591	-2	52	7.1	37	5.0	11	1.5	0.016	-14	21	2.3	77	8.8	1.9	0.2	0.003
No. 14	706	-5	50	6.8	37	5.1	13	1.8	0.017	-20	24	2.7	73	8.2	3.7	0.4	0.005
No. 19	393	19	44	6.0	44	5.9	12	1.7	0.014	-44	20	2.3	76	8.6	3.7	0.4	0.003
No. 23	334	19	46	6.2	43	5.8	11	1.5	0.014	-39	22	2.5	78	8.8	0.0	0.0	0.003
**— After 6-week incubation —**																	
Control	-	157	35	4.8	55	7.5	9.2	1.2	0.009	209	19	2.1	80	9.1	0.8	0.1	0.004
No. 2	22	128	41	5.5	52	7.1	7.1	1.0	0.013	150	20	2.3	77	8.7	3.0	0.3	0.006
No. 5	155	38	45	6.1	45	6.2	10	1.3	0.012	54	21	2.4	78	8.8	0.3	0.0	0.004
No. 9	454	17	55	7.4	35	4.8	10	1.3	0.017	-27	27	3.1	69	7.8	4.1	0.5	0.004
No. 10	239	15	50	6.8	40	5.4	11	1.4	0.012	-61	21	2.3	76	8.6	3.0	0.3	0.004
No. 13	591	-16	53	7.2	36	4.9	11	1.5	0.014	-30	27	3.1	70	7.9	3.0	0.3	0.005
No. 14	706	-5	54	7.4	36	4.8	10	1.4	0.018	-35	27	3.1	68	7.7	4.7	0.5	0.006
No. 19	393	9	52	7.1	37	5.1	10	1.4	0.014	-57	25	2.9	70	8.0	4.2	0.5	0.003
No. 23	334	-1	49	6.6	41	5.5	11	1.5	0.015	-57	26	3.0	71	8.0	2.5	0.3	0.004

ADSOM, acid-detergent soluble organic matter; As(III), arsenite; As(V), arsenate; As-S, As bound to Sulfur

R-factor, indicator of goodness of linear combination fitting (lower value indicates better fit)
